# Nanoconfinement Geometry of Pillared V_2_O_5_ Determines Electrochemical Ion Intercalation Mechanisms, Storage Sites, and Diffusion Pathways

**DOI:** 10.1021/acsnano.5c08169

**Published:** 2025-07-14

**Authors:** Jameela Karol, Charles O. Ogolla, Mohsen Sotoudeh, Manuel Dillenz, Maciej Tobis, Ellen Vollmer, Yoga T. Malik, Maider Zarrabeitia, Axel Groß, Benjamin Butz, Simon Fleischmann

**Affiliations:** † 557388 Helmholtz Institute Ulm (HIU), Helmholtzstr. 11, Ulm 89081, Germany; ‡ Karlsruhe Institute of Technology (KIT), Karlsruhe 76021, Germany; § Micro- and Nanoanalytics Group, 14312 University of Siegen, Siegen 57076, Germany; ∥ Institute of Theoretical Chemistry, 9189 Ulm University, Ulm 89081, Germany

**Keywords:** electrochemical energy storage, solvent cointercalation, bilayered vanadium pentoxide, interlayer expansion, organic−inorganic hybrid materials, electrochemical quartz crystal microbalance, ion solvation

## Abstract

Improving the electrochemical ion intercalation capacity and kinetics in layered host materials is a critical challenge to further develop lithium-ion batteries, as well as emerging cell chemistries based on ions beyond lithium. Modification of the nanoconfining interlayer space within host materials by synthetic pillaring approaches has emerged as a promising strategy; however, the resulting structural properties of host materials, host–pillar interactions as well as associated electrochemical mechanisms remain poorly understood. Herein, we systematically study a series of bilayered V_2_O_5_ host materials pillared with alkyldiamines of different lengths, resulting in tunable nanoconfinement geometries with interlayer spacings in the range of 1.0–1.9 nm. The electrochemical Li^+^ intercalation capacity is increased from approximately 1.0 to 1.5 Li^+^ per V_2_O_5_ in expanded host materials due to the stabilization of new storage sites. The intercalation kinetics improve with expansion due to a transition in Li^+^ diffusion pathways from 1D to 2D diffusional networks. Operando X-ray diffraction reveals a transition of the intercalation mechanism from solid-solution Li^+^ intercalation in V_2_O_5_ hosts with small and medium interlayer spacings to solvent cointercalation in V_2_O_5_ with the largest interlayer spacing. The work systematically demonstrates the impact of nanoconfinement geometry within bilayered V_2_O_5_ on the resulting Li^+^ intercalation metrics and mechanisms, providing insights into both the microstructure and associated electrochemistry of pillared materials.

## Graphical abstract

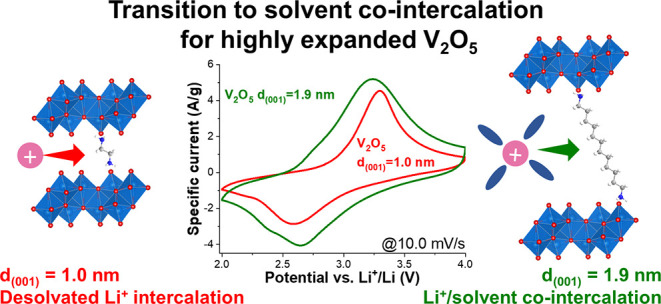

The charge storage mechanism of lithium-ion batteries, as well as of novel cell chemistries beyond lithium, is based on electrochemical ion intercalation. The process involves the reversible storage of ions in a solid-state host electrode material that is typically undergoing small structural changes. Solid-state diffusion of the ions within the host lattice, as well as associated volumetric and/or crystallographic phase changes of the host itself, can limit the kinetics of the intercalation reaction. To realize high power charge storage processes, strategies to mitigate such limitations are being developed from an electrode perspective across length scales, from the macroscopic particle size scale down to the microscopic atomic arrangement within the host.^1–4^ Vanadium oxides are widely established as host materials for ion intercalation reactions. The bilayered V_2_O_5_ phase (δ-V_2_O_5_) is particularly versatile due to its ability to host a variety of cations, such as lithium, sodium, manganese, or zinc.^5,6^ Thus, the material is suitable as a model system to explore electrochemical intercalation properties as a function of the host material structure and particularly its interlayer spacing.

Interlayer expansion of V_2_O_5_ was explored via ionic pillaring approaches.^7–9^ For example, Clites et al. studied the chemical preintercalation of different sized alkali- and alkaline-earth ions as simple ionic pillars, resulting in a variation of the interlayer spacing between 0.96 and 1.34 nm.^7^ The material with the largest initial interlayer spacing (δ-V_2_O_5_ preintercalated with Mg^2+^) was found to exhibit the highest rate behavior for the electrochemical lithiation reaction.^7^ In the tunnel-structured polymorph ζ-V_2_O_5_, preintercalation of Na^+^ or K^+^ led to an expansion of the 1D tunnels, yielding higher reversible lithium intercalation capacity and diffusivity.^9^ Several ionic pillaring approaches of δV_2_O_5_ have also been reported to improve the capacity and stability of the electrochemical Zn^2+^ intercalation reaction, demonstrating the viability of the approach also for multivalent electrochemical intercalants in an aqueous electrolyte environment.^10–12^

Beyond (monatomic/simple) ions, molecules and/or polymers have been employed as pillars to tune the nanoconfined interlayer environment of V_2_O_5_.^13,14^ Zhang et al. recently demonstrated an expansion of the interlayer spacing in bilayered V_2_O_5_ up to 3.56 nm using alkylammonium cations.^14^ Pillaring with polypyrrole or polyaniline resulted in various V_2_O_5_ phases with a widened interlayer spacing (1.4–1.5 nm) and a variety of particle morphologies.^15–18^ The studies demonstrate that the pillared materials exhibit improved electrochemical Zn^2+^ intercalation kinetics and stability compared to nonpillared V_2_O_5_, which was ascribed to “zero strain” volumetric behavior during cycling.^17^ It was also found that the bonding nature and pillar mobility influence electrochemical Zn^2+^ intercalation.^19^

Overall, the studies suggest that pillaring of V_2_O_5_ by simple ions or molecules is capable of improving capacity, kinetics, and/or stability of electrochemical ion intercalation reactions. However, there are still large gaps in the mechanistic understanding of both the structure and electrochemistry of such pillared host materials. From a materials side, the interaction between pillars and host material and associated changes in the host crystal structure upon pillar insertion are not sufficiently explored. From the electrochemistry side, there is a lack of understanding of the intercalated ions’ diffusion paths and storage sites as a function of the interlayer spacing of the host material. Systematic computational studies are required that determine the diffusion paths and associated energy barriers as a function of the host materials’ nanoconfinement geometry.^20,21^ Moreover, in highly expanded host materials, the possibility of electrolyte solvent molecules participating in the electrode reaction, i.e., the occurrence of ion–solvent cointercalation reactions,^22–25^ must be considered. This can be considered as analogous to ion desolvation effects during the electrical double-layer formation within carbon micropores of sizes below the solvated ion in supercapacitors.^23,26–28^ There is an urgent need to understand whether there can be a nanoconfinement geometry-induced evolution of the electrochemical intercalation mechanism from desolvated ions toward solvent cointercalation in pillared vanadium oxides.

To provide clear structural and mechanistic insights into molecularly pillared host materials, this work introduces a well-defined model system of a series of molecularly pillared δ-V_2_O_5_-alkyldiamine materials. These show a systematic variation in interlayer spacing (1.0–1.9 nm) owing to different lengths of alkyldiamine pillars. The model system exhibits a comparable, well-defined intralayer crystal structure where only the *c*-parameter of the host system is varied by the molecular pillars. The materials possess comparable nanowhisker-morphology, specific surface area, pillar chemistry, and pillar density in the interlayer. This allows us to link the observed electrochemical Li^+^ intercalation properties exclusively to the geometry of the nanoconfining interlayer space. We provide detailed insights into the materials’ structure and associated electrochemistry utilizing combined electrochemical operando experiments and computational investigation. By probing both, the response of the host materials, as well as the nature of intercalating species, we describe a transition from solid-solution intercalation of desolvated Li^+^ toward solvent cointercalation in the V_2_O_5_ electrode with the largest interlayer expansion. Moreover, the work demonstrates the stabilization of new Li^+^ storage sites and a transformation from 1D to 2D diffusional networks in expanded V_2_O_5_, increasing both capacity and kinetics of the lithiation reaction.

## Results and Discussion

### Structural Investigation

The goal of this work is to unambiguously link the nanoconfinement geometry of a layered host electrode material, bilayered V_2_O_5_, with the resulting electrochemical ion intercalation properties. For this purpose, model electrode materials with different, well-defined interlayer spacings are synthesized by employing alkyldiamines of various lengths that act as molecular pillars/spacers within the interlayer. As illustrated in Figure 1A, ethylenediamine (2C-DA), 1,6-hexanediamine (6C-DA), or 1,12-dodecanediamine (12C-DA) are dissolved in water (water/ethanol mixture in case of 12C-DA for better solubility) together with commercially available α-V_2_O_5_ powder and undergo hydrothermal treatment at 100 °C for 12 h in an autoclave. For further comparison, a reference sample with preintercalated Li^+^ and H_2_O (Li–V_2_O_5_) is synthesized via a sol–gel process followed by hydrothermal treatment, as described by Clites et al.^7^ (Figures 1A and S1).

**Figure 1:**
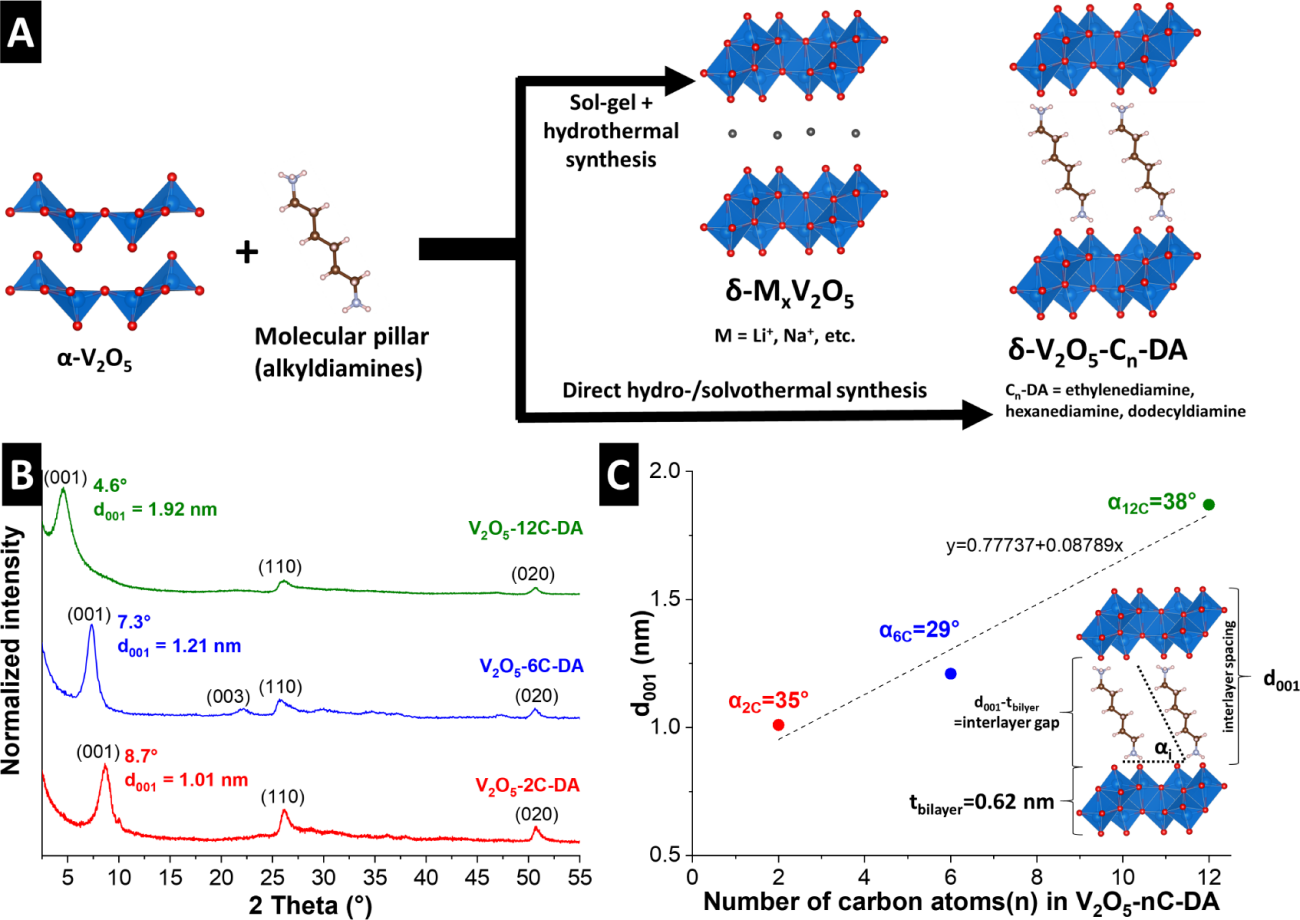
(A) Illustration of the synthesis procedure of alkyldiamine-pillared, bilayered V_2_O_5_ samples. (B) X-ray diffractograms (Cu Kα, λ = 1.5406 Å) of the samples. Data is normalized to the highest peak intensity. (C) interlayer spacing calculated from the 2θ position of the (001) reflection as a function of the number of carbon atoms in the used alkyldiamine pillar, including linear fit. Inset of bilayered V_2_O_5_ structure illustrates geometrical relation between (crystallographic) interlayer spacing (d_001_) from XRD, interlayer gap size and the tilt angle α_*i*_ of the pillars, assuming a linear conformation of the molecules.

Figure 1B shows the powder XRD patterns of the alkyldiamine-pillared vanadium oxides used to determine the crystal structure and interlayer spacing of the synthesized materials. The diffractograms indicate that all three alkyldiamine-pillared V_2_O_5_ samples exhibit the main characteristic peaks corresponding to bilayered δ-V_2_O_5_, according to the structural model proposed by Petkov et al.^29^ This includes peak maxima at 26.1° and 50.7° 2θ, related to the (110) and (020) reflections, respectively. The visibility of the (003) reflection for V_2_O_5_-6C-DA indicates that this sample has the highest structural order among the three samples. The different (001) diffraction angles of the three samples located at 8.7° (2C-DA), 7.3° (6C-DA), and 4.6° 2θ (12C-DA), demonstrate the successful tailoring of the interlayer spacing of V_2_O_5_ bilayers using alkyldiamine pillars of different lengths. The corresponding (001) spacings derived from XRD are 1.01 nm (2C-DA), 1.21 nm (6C-DA) and 1.92 nm (12C-DA). Li–V_2_O_5_ synthesized via the sol–gel route exhibits the same crystal structure with a (001)-spacing of 1.22 nm (Figure S1B), which is in the typical range of bilayered V_2_O_5_ materials with nanoconfined water and/or alkali cations.^7,30^ Overall, XRD results demonstrate that all pillared V_2_O_5_ samples exhibit relatively low crystalline order and/or nanocrystalline domain sizes. This is also the reason for signals in the region between ca. 28–50° 2θ, which are hard to unambiguously correlate with particular sets of planes of the bilayered V_2_O_5_ structure due to their very low intensity. Therefore, we employ further crystal structural analysis of the bilayered V_2_O_5_ host structure on a nanoscale by highly localized transmission electron imaging/diffraction techniques, vide infra.

Linear fitting of the measured (001) spacings over the number of carbon atoms in the pillars’ alkyl-chains demonstrates a positive correlation (Figure 1C). The arrangement of alkyldiamine pillars within the V_2_O_5_ host structure is approximated by geometrical considerations (Figure 1C, inset), in accordance with previous work on pillared layered/2D materials.^31,32^ These approximations are based on the assumptions that (1) most alkyldiamines interact with V_2_O_5_ bilayers at both opposing sides of the interlayer galleries via their functional amine/ammonium groups, i.e., form “bridges” between the layers as described for insertion of alkyldiamines into clays under acidic conditions.^33^ Further, (2) the alkyl-chains are assumed in a straight conformation (not bent or twisted), and (3) the molecule lengths are approximated by their van der Waals radii given an electrostatic pillar-host interaction. With alkyldiamine molecular lengths of 0.67 nm (2C-DA), 1.22 nm (6C-DA), and 2.1 nm (12C-DA) and a V_2_O_5_ bilayer thickness of 0.62 nm, the molecules are assumed to assemble with tilt angles α_*i*_ of 35°, 29°, and 38°, respectively, with respect to the bilayer direction (Figure 1C, inset). It should be noted that the crystallographic interlayer spacing (obtained, for example, from XRD) does not correspond to the interlayer gap size, as further illustrated in Figure 1C.

An in-depth correlative microscopy investigation of the synthesized samples with variable nanoconfinement geometry is undertaken to elucidate the pillar-induced changes in the host structure. Scanning and transmission electron microscopy (SEM and TEM) are leveraged to confirm the materials’ homogeneity, to gain insights into their morphology, to assess crystallinity and to verify the crystal structure (Figure 2). All the molecularly pillared samples (V_2_O_5_-2C-DA, V_2_O_5_-6C-DA and V_2_O_5_-12C-DA) show characteristic nanowhisker morphology (SEM: Figure 2A–C, TEM: Figure 2D-F), allowing for individual free-standing strands to be investigated further. The V_2_O_5_-12C-DA system shows stronger bundling/agglomeration of the whiskers (Figure 2C,F) compared to V_2_O_5_-2C-DA and V_2_O_5_-6C-DA. This is attributed to the solvent modification (ethanol–water mixture instead of only water, which is necessary to adequately dissolve 1,12-dodecanediamine).^34,35^ TEM bright-field imaging (Figure 2D–F) verifies the sample homogeneity and single-crystalline nature of the whiskers with low defect density. The nanowhiskers of high aspect ratio are found to be a few tens of nanometers wide and a few up to tens of micrometers long. Overall, the nanowhisker morphology of all samples is similar, but shows the highest homogeneity for V_2_O_5_-6C-DA.

**Figure 2:**
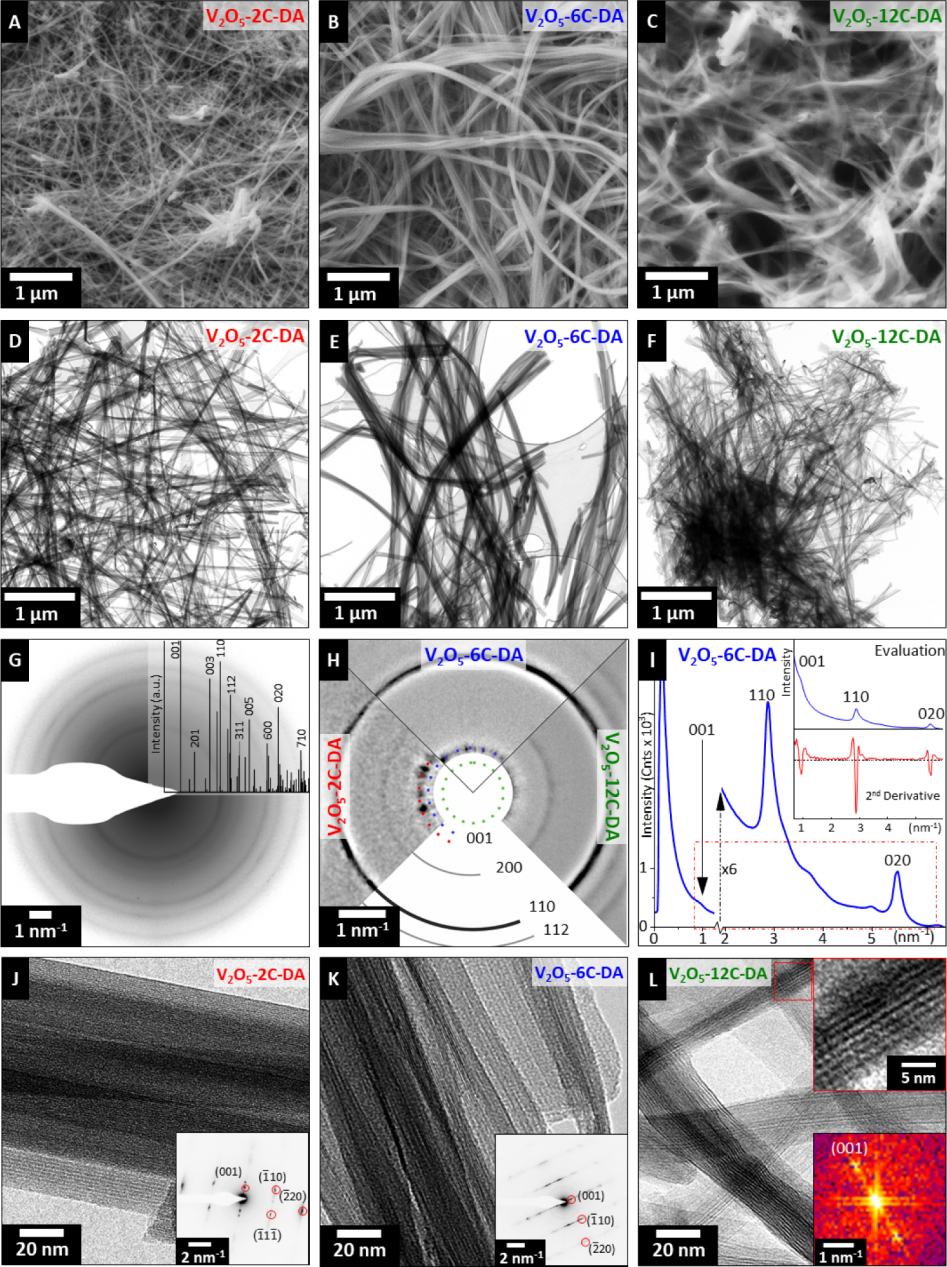
Morphology, microstructure, and crystal structure analyses by SEM and TEM: SEM images of (A) V_2_O_5_-2C-DA, (B) V_2_O_5_-6C-DA and (C) V_2_O_5_-12C-DA; (D)–(F) respective BFTEM images; (G) representative powder SAED (logarithmic display) of V_2_O_5_-12C-DA with simulation (structural model by Petkov et al.^29^ confirming the high degree of crystalline order; (H) comparison of SAED pattern centers (logarithmic display) with molecular spacer-dependent rings of (001) reflections in relation to the constant (200)/(110) rings; (I) quantitative determination of (001) spacings from patterns in (H) by azimuthal averaging (radial intensity plot) and peak background correction by second-order derivative calculation; (J)–(L) representative HRTEM micrographs with corresponding single-crystal SAED patterns (insets) of individual wires. In (L), the power spectrum (FFT) is displayed instead due to the (001) peak being located in close vicinity to the beam blocker.

While sharing an unambiguous nanowhisker morphology with the (001) basal planes being systematically aligned parallel to the long whisker dimension (Figure 2J–L), significant differences in interlayer spacing are revealed between the samples, attributed to the successful pillaring of the bilayered V_2_O_5_ host structure using alkyldiamine of three lengths. This is concluded from powder X-ray diffraction (XRD), selected-area electron diffraction (SAED) and high-resolution TEM (HRTEM) analyses (Figures 1B and 2G–L, respectively). Complementary to the XRD, the crystal structure of the samples is confirmed by powder SAED of representative whisker ensembles. All three samples present a monoclinic crystal structure of the space group *C2*/*m* characteristic of such bilayered δ-V_2_O_5_ systems.^29^ Comparing simulated with measured SAED patterns of the three samples, it becomes clear that a systematic increase in the (001) lattice spacing from 0.95 to 1.10 nm is observed for 2C-DA and 6C-DA, respectively (Figure 2G,H). The (001) lattice spacing of 12C-DA cannot be accessed via SAED because of its proximity to the beam blocker. It is therefore derived from fast Fourier transform (FFT, Figure 2L inset). In all three samples, the prominent SAED signals corresponding to (020) and (110) planes are identical, demonstrating no pillar-induced alteration of in-plane host structure of the bilayered V_2_O_5_ phase (Figure 2H). It should be noted that the smaller (001)-spacing derived from TEM analysis compared to XRD analysis likely results from partial pillar degradation under the high energy electron beam and/or loss of confined interlayer species under vacuum conditions, although measures like minimal-dose imaging/diffraction were applied.^36,37^ Nonetheless, the basal planes are continuous, well aligned and extend along the whole whisker. Moreover, the interlayer spacing within individual whiskers appears to be constant. Extended defects like kinks, cracks, discontinuities, grain boundaries or dislocations are rarely observed (Figure 2D–F,J–L) proving the high quality of the synthesized materials. The *c*-parameters obtained from SAED are confirmed by localized HRTEM (insets of Figure 2J–L). The results confirm that the employed alkyldiamine pillaring approach successfully leads to a variation of the V_2_O_5_ nanoconfinement geometry, while no changes in intralayer structure and morphology are observed.

The pillar content of the samples is analyzed by means of thermogravimetric analysis (TGA). Heating to 550 °C under a constant oxygen flow leads to the thermal decomposition and/or burn-off of any organic components in the V_2_O_5_ samples (caution: temperatures above ca. 600–650 °C should be avoided in these TGA experiments due to the low melting temperature of V_2_O_5_). The mass loss upon heating of the V_2_O_5_-alkyldiamine samples occurs mainly in two steps, as shown in Figure 3A. The mass loss below ca. 150 °C is attributed to the loss of surface water and/or crystal water in the interlayer space.^38^ This mass loss corresponds to ca. 5, 3.5, and 2 wt % for V_2_O_5_-2C-DA, V_2_O_5_-6C-DA, and V_2_O_5_-12C-DA, respectively. The second step between ca. 200–400 °C, accounting for a subsequent mass loss of 8, 9.7, and 16.5 wt % for V_2_O_5_-2C-DA, V_2_O_5_-6C-DA, and V_2_O_5_-12C-DA, respectively, is ascribed to the decomposition and/or burn-off of the alkyldiamine pillars.^39^ This leads to calculated chemical compositions of the samples as V_2_O_5_-(2C-DA)_0.29_, V_2_O_5_-(6C-DA)_0.18_, and V_2_O_5_-(12C-DA)_0.19_. While the molar ratio of educts in the reactions is chosen as 1:1 for alkyldiamine to vanadium, the TGA experiments reveal a product composition with significantly fewer pillars. The ratio of alkyldiamine pillars in the products is comparable for all samples, with a slightly higher number for 2C-DA, which is explained by its small size and low boiling point that allows for higher mobility during the synthesis.

**Figure 3:**
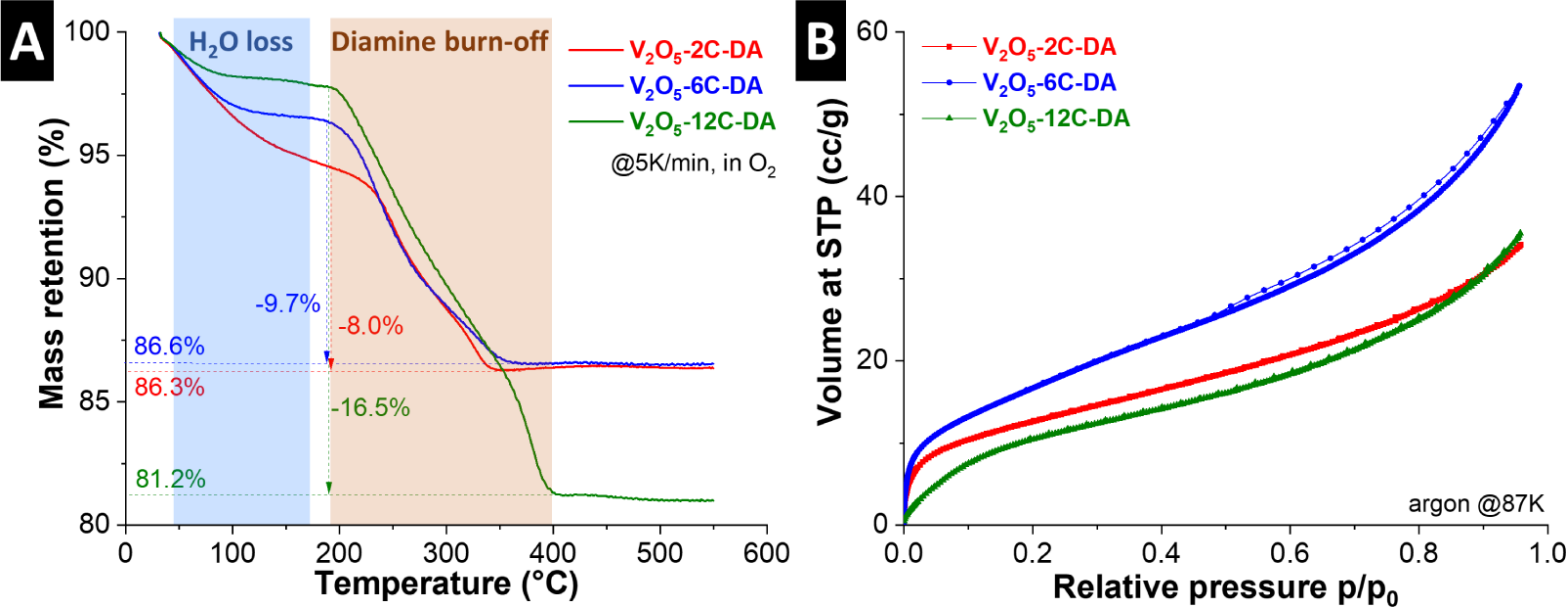
(A) Thermogravimetric analysis (TGA) of all samples in oxygen atmosphere at a heating rate of 5 K/min. (B) Argon sorption isotherms of all samples measured at 87 K (STP = standard temperature and pressure).

To analyze the specific surface area and porosity of the samples, argon gas sorption measurements (GSA) are performed as shown in Figure 3B, and the specific surface area of the samples is calculated using the Brunauer–Emmett–Teller (BET) equation.^40^ The BET surface area is calculated to be 40 m^2^/g for V_2_O_5_-2C-DA, 57 m^2^/g for V_2_O_5_-6C-DA and 37 m^2^/g for V_2_O_5_-12C-DA. All sorption isotherms exhibit a Type II shape without significant hysteresis, according to IUPAC classification, indicating a nonporous or macroporous character of all samples.^41^ It is notable that the expanded interlayer space, even in the case of V_2_O_5_-12C-DA, appears inaccessible for gas sorption. The GSA analysis confirms a comparable surface area and porosity for all samples.

The interaction between the pillaring alkyldiamine molecules and the V_2_O_5_ host is analyzed by X-ray photoelectron spectroscopy (XPS). Both the O 1s and V 2p signals of all alkyldiamine-pillared samples are shown in Figure 4A–C. Two signals corresponding to the spin–orbit of V 2p_3/2_ and V 2p_1/2_ are located in the energy ranges around 517 and 525 eV, respectively, which contain contributions of V­(V) and V­(IV), in agreement with the highly intense peak in the O 1s.^42,43^ This shows that surface vanadium in the alkyldiamine-pillared V_2_O_5_ samples is partially reduced after the chemical synthesis. In addition, the O 1s region shows a broad shoulder at higher binding energies, corresponding to carbon–oxygen species (−C=O, −COC– and/or −COH) from surface reactivity in ambient atmosphere. The N 1s signals (Figure 4D–F) show the main signal centered at 401.5 eV for V_2_O_5_-2C-DA, 401.2 eV for V_2_O_5_-6C-DA, and 401.8 eV for V_2_O_5_-12C-DA, which can be assigned to a positively charged nitrogen, such as −NH_3_^+^.^44^ Hence, most pillar functional groups form cationic ammonium groups in the aqueous reaction solution, which interact by ionic bonding with the V_2_O_5_ host. The main N 1s signal of the three samples displays a shoulder at a lower binding energy of 399.3 eV that has been described for −C–NH_2_,^45,46^ indicating the simultaneous presence of small amounts of neutral alkyldiamine, which can be assumed as noninteracting with the V_2_O_5_ host via ionic or covalent bonds. Overall, XPS results demonstrate the predominantly ionic interaction between the partially reduced V_2_O_5_ host and cationic ammonium groups, while a small residue of noninteracting amine groups is also found.

**Figure 4:**
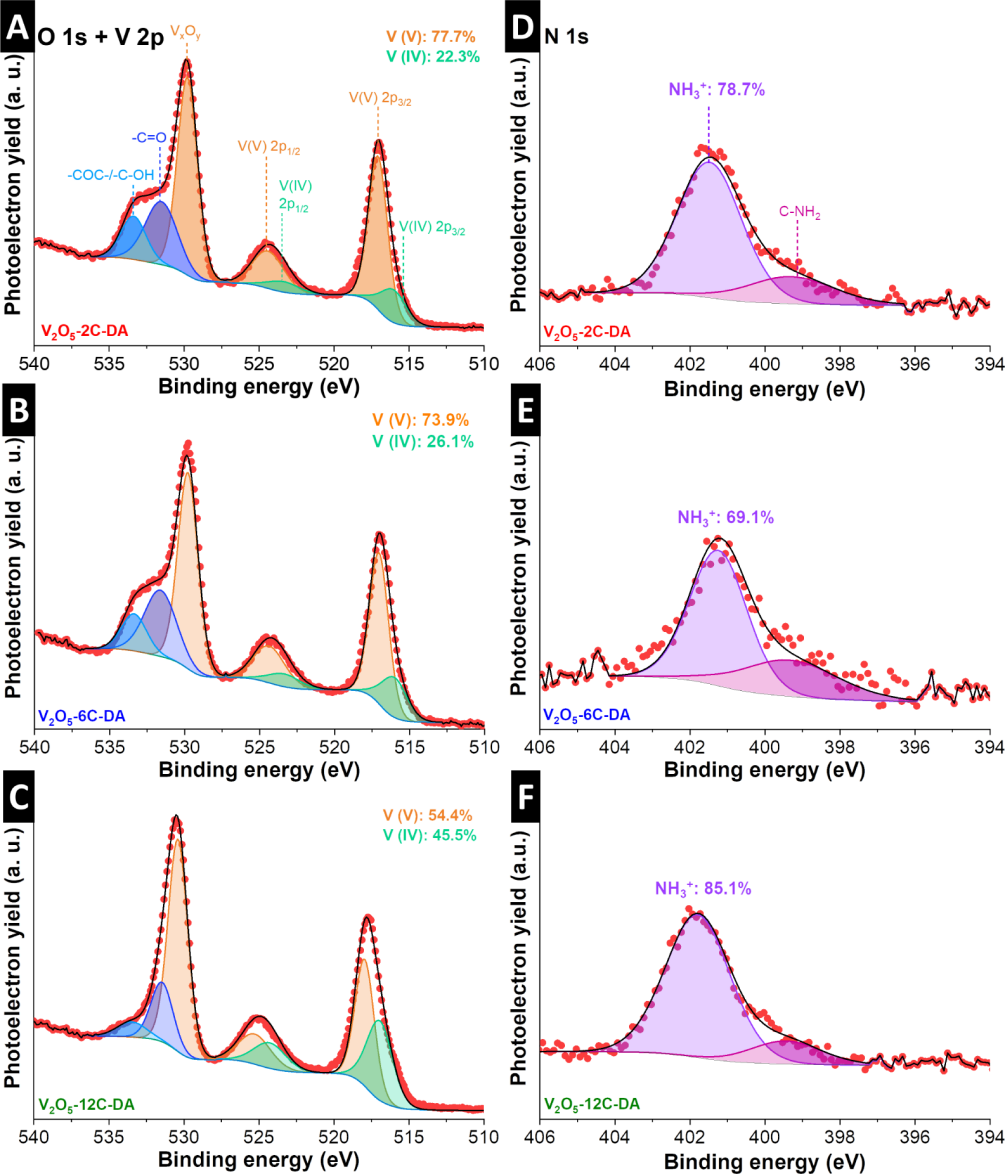
O 1s, V 2p and N 1s photoelectron lines of (A, D) V_2_O_5_-2C-DA, (B, E) V_2_O_5_-6C-DA and (C, F) V_2_O_5_-12C-DA.

### Electrochemical Characterization

Bilayered V_2_O_5_ materials are synthesized with three different nanoconfinement geometries by using alkyldiamine pillars with variable lengths. The structural investigation demonstrates that they exhibit highly comparable morphology, surface area, intralayer crystal structure, density of pillaring molecules in the interlayer, and ionic pillar–host interaction. Hence, they are suitable as model electrode materials to analyze the impact of the nanoconfinement geometry/interlayer spacing on the electrochemical Li^+^ intercalation reaction, because influences of other structural features on the electrochemical signal remain negligible across the three samples.

Galvanostatic charge/discharge (GCD) profiles at rates between 20 and 5,000 mA/g are shown in Figure 5A–C. The potential profiles at low rates are comparable for all samples, with variations in the maximum capacity. The samples show cathodic (lithiation) capacities at a rate of 20 mA/g with 169 mAh/g (V_2_O_5_-2C-DA), 193 mAh/g (V_2_O_5_-6C-DA) and 155 mAh/g (V_2_O_5_-12C-DA). However, at higher rates like 5 A/g, a reduced polarization is observed for V_2_O_5_-12C-DA compared to V_2_O_5_-2C-DA and V_2_O_5_-6C-DA, indicating improved kinetics for the V_2_O_5_ host with the largest interlayer spacing. It should be noted that the reported values of specific capacity take into account the mass of both the V_2_O_5_ host and the alkyldiamine pillars, where the latter do not contribute to the reversible charge storage process. Thus, to gain fundamental insights into the charge storage process in pillared V_2_O_5_ hosts, quantification of stored Li^+^ per structural unit of V_2_O_5_ is derived from the reversible, electrochemically stored charge and the quantity of pillars derived from TGA. Calculation of the maximum number of Li^+^ stored yields stoichiometries of Li_1.02_V_2_O_5_, Li_1.27_V_2_O_5_-2C-DA, Li_1.48_V_2_O_5_-6C-DA, and Li_1.33_V_2_O_5_–12C-DA, demonstrating increased storage capacity of pillared V_2_O_5_ despite the additional presence of pillar molecules in the interlayer space.

**Figure 5:**
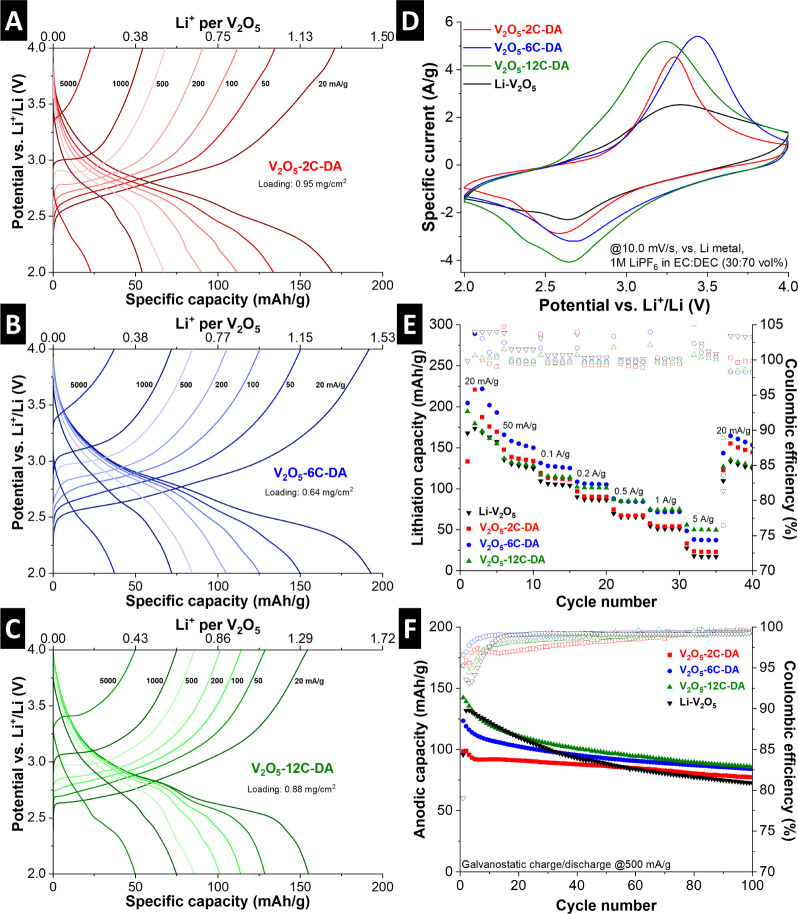
Galvanostatic charge/discharge profiles of (A) V_2_O_5_-2C-DA, (B) V_2_O_5_-6C-DA, (C) V_2_O_5_-12C-DA, at specific currents of 20, 50, 100, 200, and 500 mA/g. Li^+^ per V_2_O_5_ refers to the storage capacity with respect to V_2_O_5_ in the respective sample, omitting mass of molecular pillar. (D) Cyclic voltammograms of Li–V_2_O_5_, V_2_O_5_-2C-DA, V_2_O_5_-6C-DA and V_2_O_5_-12C-DA at a sweep rate of 10.0 mV/s. (E) Cathodic capacity from GCD for 5 cycles each at specific currents of 20, 50, 100, 200, 500, 1000, and 5000 mA/g. (F) Long-term GCD at 500 mA/g. All measurements are conducted in coin cells versus Li metal electrodes in 1 M LiPF_6_ in EC/DEC (30:70 vol %) electrolyte. All mass-normalizations are with respect to the mass of the full V_2_O_5_-alkyldiamine composite.

Cyclic voltammograms (CVs) at a rate of 10 mV/s give additional insights into the (de)­lithiation process (Figure 5D). While all (cathodic) lithiation peaks are roughly centered at around 2.6–2.7 V vs Li^+^/Li, the peak shape becomes significantly broader for V_2_O_5_-12C-DA, with a small additional feature at around 2.3 V, indicative of a change in lithiation mechanism for V_2_O_5_ with the largest interlayer spacing. Furthermore, the overpotential is reduced for V_2_O_5_-12C-DA, which shows the onset of the (anodic) delithiation peak at lower potentials compared to V_2_O_5_-2C-DA and V_2_O_5_-6C-DA, demonstrating improved electrochemical reversibility for V_2_O_5_-12C-DA at such a high sweep rate. Comparison with the CV of a bilayered Li–V_2_O_5_ reference sample shows significantly improved electrochemical reversibility, indicating improved kinetics for alkyldiamine-pillared samples compared to preintercalated Li^+^.

Quantitative analysis of the capacity at different currents is probed by GCD at rates up to 5 A/g (Figure 5E). The trends of improved kinetics for larger interlayer spacings are reflected in the capacity retention at higher rates, with V_2_O_5_-12C-DA showing the highest capacity retention of 50 mAh/g compared to 37 mAh/g (V_2_O_5_-6C-DA) and 23 mAh/g (V_2_O_5_-2C-DA) at a rate of 5 A/g.

The cycling stability of pillared V_2_O_5_ materials is probed by GCD at 0.5 A/g over 100 cycles and shown in Figure 5F. All samples show a significant initial capacity decay over ca. 5–10 cycles and a slow but linear capacity reduction over the next 90 cycles, which is in line with other reports on lithium (de)­intercalation in V_2_O_5_.^7,9^ To probe whether the capacity decay is a consequence of the V_2_O_5_ host structure or disintegrating pillaring structure, ex situ XRD measurements of electrodes after 50 cycles are presented (Figure S3). The diffractograms show that the initial interlayer spacing is retained, demonstrating that alkyldiamine pillars remain in the V_2_O_5_ structure. Consequently, future work with a focus on practical application will include the transfer of this pillaring approach to host systems that exhibit higher intrinsic cycling stability than bilayered V_2_O_5_, which is known for a limited long-term stability of Li^+^ intercalation.^7^

Electrochemical impedance spectroscopy (EIS) of the pillared V_2_O_5_ electrodes is measured at various states of charge, i.e., at open circuit voltage (OCV), at the onset and at the peak potential of the lithiation process (Figure 6). At OCV, the Nyquist plots of all three samples exhibit a semicircle in the midfrequency range and a sloping response in the low-frequency range. With progressive lithiation at lower electrode potentials, an increasing impedance contribution in the low-frequency region is observed for V_2_O_5_-2C-DA and V_2_O_5_-6C-DA (Figure 6A,B), with typical Warburg-behavior indicative of semi-infinite diffusion limitation. In the midfrequency region, these electrodes show the evolution of another semicircle at 2.6 V vs Li^+^/Li, indicative of significant charge transfer resistance with interfacial processes. Contrarily, V_2_O_5_-12C-DA exhibits progressively reduced impedance in the low-frequency range with increasing lithiation (Figure 6C), indicative of improved solid-state diffusion kinetics compared to V_2_O_5_-2C-DA and V_2_O_5_-6C-DA. Moreover, the second semicircle at 2.6 V is significantly reduced in magnitude, indicating reduced charge transfer resistance. The observations indicate improved kinetics of V_2_O_5_-12C-DA and are in alignment with the electrode material’s highest capacity retention at high GCD currents.

**Figure 6:**
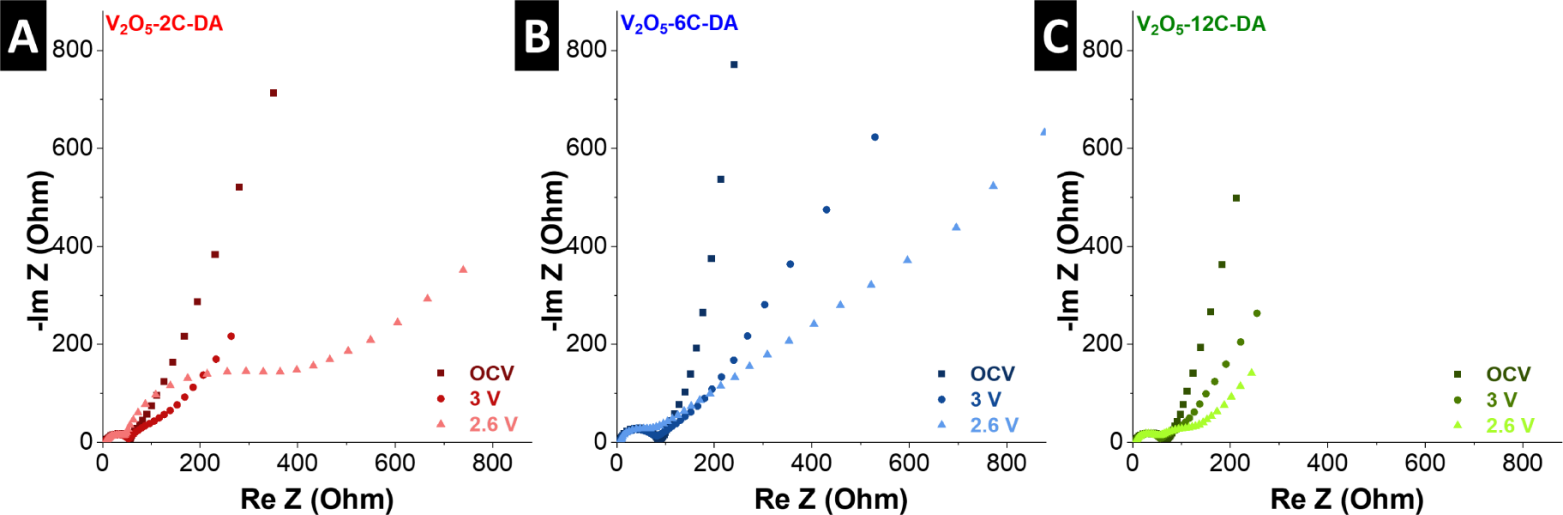
Electrochemical impedance analysis at varying states of charge of (A) V_2_O_5_-2C-DA, (B) V_2_O_5_-6C-DA and (C) V_2_O_5_-12C-DA. Measured in three-electrode cells with separate Li quasi-reference electrode.

The EIS results on transport kinetics are also in line with b-value analysis using CVs at varying rates, where a b-value of 0.5 corresponds to semi-infinite diffusion-limited and a b-value of 1 to surface-limited intercalation kinetics (Figure S4).^47,48^ V_2_O_5_-2C-DA and V_2_O_5_-6C-DA exhibit comparable b-values around 0.69–0.73 for the (de)­lithiation processes, whereas V_2_O_5_-12C-DA exhibits higher values around 0.79–0.80 indicative of improved Li^+^ transport kinetics.

### Mechanistic Investigation

To gain mechanistic insights into the electrochemical (de)­lithiation process as a function of the nanoconfinement geometry of bilayered V_2_O_5_, we carry out a series of electrochemical operando experiments to understand both the structural evolution of the host material using a combination of operando XRD and electrochemical dilatometry (ECD), as well as identify the nature of the intercalating species using electrochemical quartz crystal microbalance (EQCM).

The evolution of the (001) *d*-spacing of the electrode materials is studied by operando XRD over five consecutive cycles. For V_2_O_5_-2C-DA (Figure 7A), at open circuit potential (OCP, ca. 3.5 V vs Li^+^/Li) the (001) signal is located at 4.0° 2θ (Mo Kα source), corresponding to a *d*-spacing of 1.02 nm in line with the pristine V_2_O_5_-2C-DA material. This excludes the possibility of spontaneous ion and/or solvent insertion into the host material that is not driven by an external electrochemical stimulation. Upon electrochemical reduction (lithiation) to 2.0 V vs Li^+^/Li and subsequent oxidation (delithiation) to 4.0 V vs Li^+^/Li, the position of the (001) signal continuously shifts with maxima between ca. 3.96° 2θ (lithiated) and 4.02° 2θ (delithiated). This indicates lithium (de)­intercalation via a solid-solution mechanism associated with an almost negligible variation of *d*-spacing between 1.03 and 1.01 nm.

**Figure 7:**
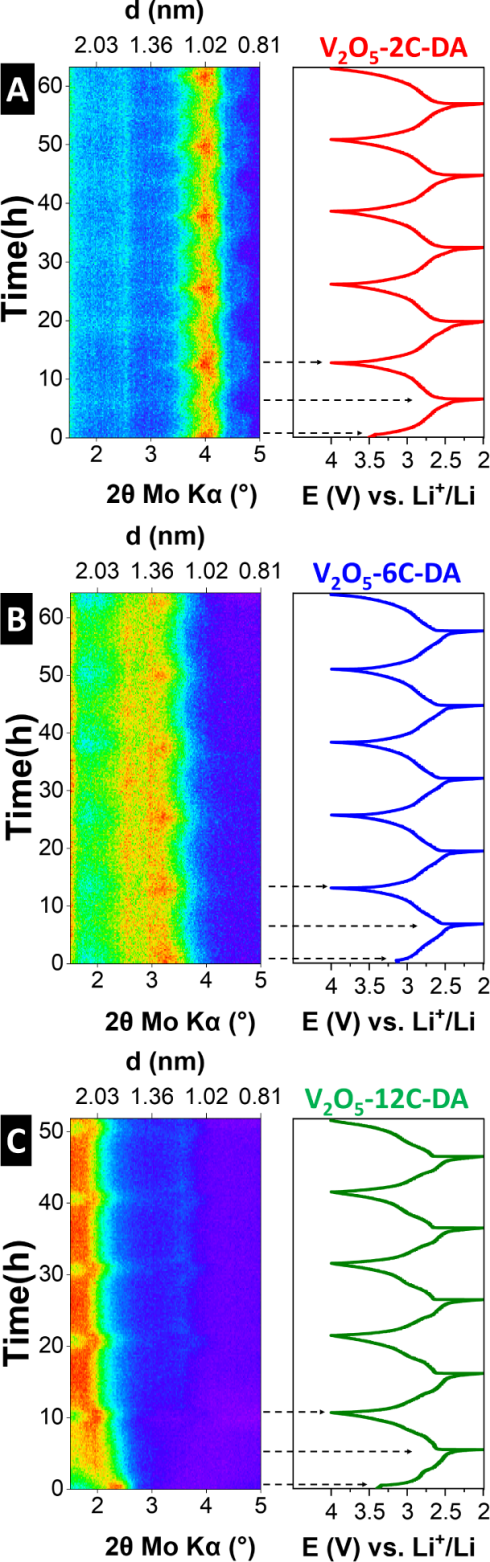
Electrochemical (de)­lithiation mechanism studied via operando XRD (Mo Kα X-ray source, λ = 0.71 Å) using a Debye–Scherrer (transmission) geometry in modified coin cells versus lithium metal over five consecutive galvanostatic charge/discharge cycles at a specific current of 50 mA/g. (A) V_2_O_5_-2C-DA, (B) V_2_O_5_-6C-DA, and (C) V_2_O_5_-12C-DA.

For V_2_O_5_-6C-DA (Figure 7B), comparable behavior is observed with the position of the (001) signal at OCP centered at ca. 3.3° 2θ, corresponding to a *d*-spacing of 1.23 nm in line with the pristine V_2_O_5_-6C-DA material. At the same time, reversible and continuous shifts upon electrochemical reduction and oxidation to ca. 1.28 and 1.25 nm are observed, respectively, suggesting the same solid-solution lithium (de)­intercalation mechanism as in V_2_O_5_-2C-DA. In both cases, the Li^+^ intercalation-induced *d*-spacing changes of pillared V_2_O_5_-2C-DA and V_2_O_5_-6C-DA are very small and highly reversible over 5 consecutive cycles.

For V_2_O_5_-12C-DA (Figure 7C), differences in the structural evolution of the host material are observed. At OCP, the material exhibits a (001) signal centered at ca. 2.3° 2θ corresponding to a *d*-spacing of 1.77 nm, which is slightly lower than that of the pristine V_2_O_5_-12C-DA material. During lithiation, an initial, abrupt increase in *d*-spacing to 2.04 nm is observed, followed by a further, more continuous increase to about 2.48 nm in the fully lithiated state at 2.0 V. Upon subsequent delithiation, contraction of the (001) *d*-spacing to 2.07 nm is observed, and the behavior is highly reversible in the subsequent cycles. The abrupt expansion of the host during the first electrochemical reduction is indicative of significant structural changes induced by the intercalation of a large guest species. Such behavior is reminiscent of Li^+^-solvent cointercalation behavior, for example, in Ti_3_C_2_T_*x*_ MXene host electrodes,^49,50^ or in pillared hydrogen titanates.^25^ We hypothesize that in the present case of V_2_O_5_-12C-DA, the large initial *d*-spacing allows for such an effect with solvent molecules from the electrolyte entering the interlayer space together with Li^+^.^23^

While operando XRD demonstrates strong expansion of the V_2_O_5_-12C-DA upon electrochemical reduction on a crystallographic level, the behavior is verified on a macroscopic level using complementary electrochemical dilatometry (ECD) experiments (Figure 8).^51^ We compare V_2_O_5_-6C-DA as a host material representative of solid-solution intercalation behavior of desolvated Li^+^ with V_2_O_5_-12C-DA, the host material where we suspect the cointercalation mechanism of (partially) solvated Li^+^. We find significant electrode expansion in V_2_O_5_-12C-DA (ca. 8%) compared to electrode contraction in V_2_O_5_-6C-DA (ca. −2.5%) upon the initial electrochemical reduction. In subsequent cycles, the much larger magnitude of V_2_O_5_-12C-DA electrode height changes compared to V_2_O_5_-6C-DA persists, providing further support for the nanoconfinement geometry-induced change to solvent cointercalation in V_2_O_5_-12C-DA. It should be noted that an overall contraction of electrode height in the delithiated state for both materials can be explained by electrode densification, considering the porous character of the electrode on a macroscopic scale.

**Figure 8:**
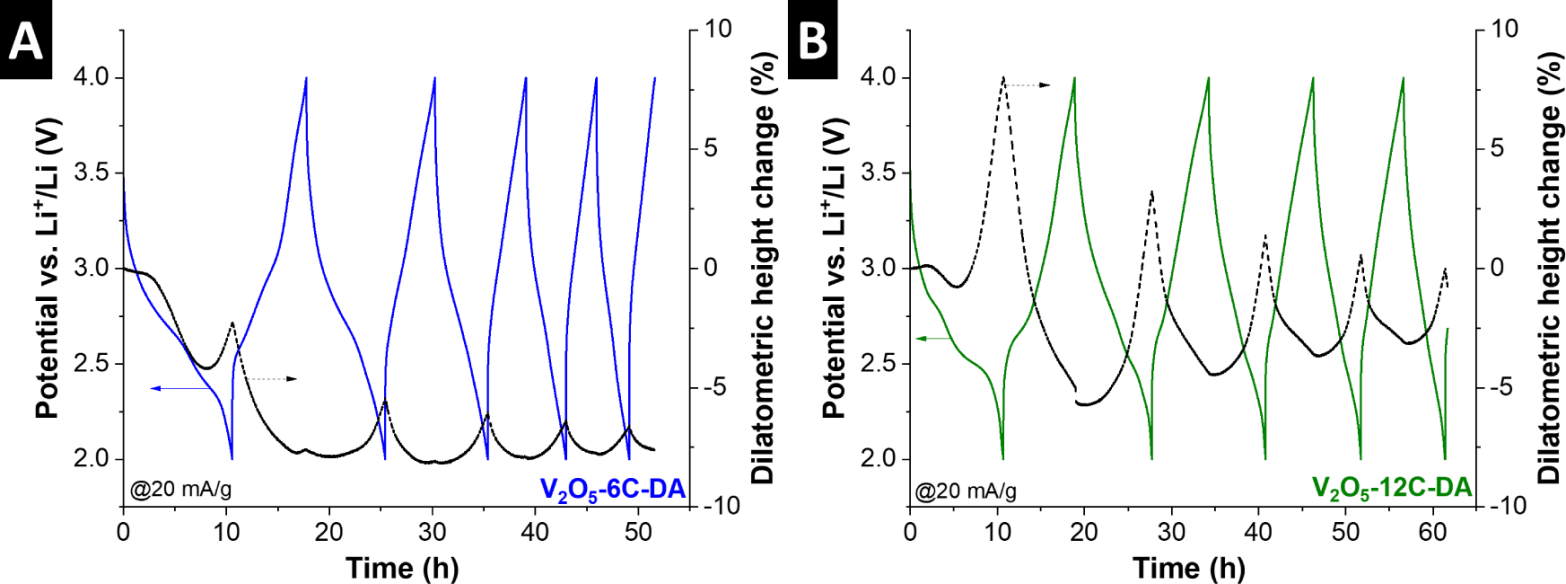
Electrochemical dilatometry of (A) V_2_O_5_-6C-DA and (B) V_2_O_5_-12C-DA over five consecutive galvanostatic (de)­lithiation cycles at 20 mA/g.

The hypothesized cointercalation mechanism in V_2_O_5_-12C-DA involves the insertion of solvent molecules from the electrolyte into the host material. It would result in a significant increase in mass change per inserted Li^+^ compared to the solid-solution intercalation of desolvated Li^+^. This is verified with EQCM measurements, which probe the resonance frequency change and dissipation of an electrode coating on a quartz resonator during electrochemical cycling. The method makes use of the linear relationship between frequency change and mass change of rigid (thin-film) coatings, allowing us to assess the mass of the intercalating species.^52^ We compare EQCM measurements of V_2_O_5_-6C-DA with V_2_O_5_-12C-DA, confirming that both electrode coatings exhibit mostly rigid properties in the dry state. They provide stable electrochemical signals over several cycles that closely align with the data obtained in coin cells (Figure S5). Both electrodes show decreasing frequency during lithiation and increasing frequency during delithiation (Figure 9A,B); however, the magnitude of these changes differs significantly. When plotting the frequency changes of both electrodes over the cumulative charge during the lithiation step, we find about an order of magnitude stronger frequency changes for V_2_O_5_-12C-DA per electron transfer. When assuming a purely gravimetric regime (i.e., no dissipation), the molecular weight of the intercalating species per electron transfer can be estimated from the slope of the plots of electrode mass change versus charge (Figure 9C). For V_2_O_5_-6C-DA, such an estimation leads to a molecular weight of ca. 20 g/mol in the linear region of the reduction step, indicative of desolvated Li^+^. Note that this value can also be increased due to the accumulation of solvent molecules at the electrochemical interface.^53^ In comparison, the molecular weight per electron transfer in V_2_O_5_-12C-DA is estimated to be around 190 g/mol, which is significantly higher and indicative of the cointercalation of carbonate solvent.

**Figure 9:**
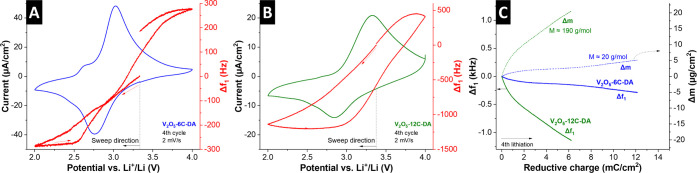
EQCM measurements of (A) V_2_O_5_-6C-DA and (B) V_2_O_5_-12C-DA with cyclic voltammograms at 2 mV/s and simultaneously monitored (fundamental) frequency change (Δf_1_) within the cycle. (C) Cumulative charge from integration of the current over time during the reduction step plotted against the measured frequency change and the calculated mass change assuming validity of the Sauerbrey equation (purely gravimetric regime). The molecular weight (M) of the intercalating species per electron transfer is estimated from the slopes of the linear regions.

To evaluate the resonance properties of the spray-coated films of active materials, we monitor several overtones (*n* = 3, 5, 7, 9, 11) and find that the related frequency changes (*Δf*_*n*_/*n*) are not fully independent of the overtone over the entire cycling range and that dissipation changes exceeding 100 ppm develop (Figure S6). This indicates that viscoelastic properties emerge during electrochemical cycling,^54^ hence, the estimated mass changes via the Sauerbrey equation should only be interpreted qualitatively. Precise quantification requires a fully elastic regime during electrochemical cycling, which may be achieved by optimizing the active material coating, for example, through the selection of different binders and/or monodisperse particle sizes.^54^

### Computational Investigation

First-principles calculations within the framework of Density Functional Theory (DFT) are conducted to gain a deeper understanding of the thermodynamics and kinetics involved in the Li^+^ intercalation process in bilayered V_2_O_5_ intercalation hosts as a function of their nanoconfinement geometry, i.e., interlayer spacing. In our preliminary model construction, we adopt the crystal structure observed for V_2_O_5_·*n*H_2_O again according to the model of Petkov et al.^29^ As described in the structural characterization section, this structure exhibits bilayers of V_2_O_5_ arranged in a stacking fashion along the *c*-axis within a monoclinic unit cell (space group *C*2/*m*). These layers are composed of square pyramidal VO_5_ units as shown in Figure S6A,B. The interlayer distance expands or contracts as the guest ions or molecules intercalate within the bilayered framework.

The first step is the construction of an intercalant-free interlayer structure, with subsequent optimization based on DFT calculations, resulting in 0.87 nm interlayer spacing, and an interlayer gap between the two individual layers of V_2_O_5_ of approximately 0.29 nm. Details on the precise unit cell parameters utilizing different functionals are provided in Table S1.

The impact of increased interlayer spacing on Li^+^ diffusion pathways in pillar-free V_2_O_5_ was examined using the Bond Valence Site Energy (BVSE) approach as shown in Figure S7. According to the static BVSE model, there are 1D diffusion pathways for Li^+^ sites with interlayer spacing of 0.87 nm (Figure S7A). However, an increase in interlayer spacing facilitates the connection between channels, resulting in the formation of a 2D network of Li^+^ pathways. Interestingly, the diffusion pathways for the system with an increase in interlayer spacing to 1.21 nm illustrate interactions with oxygen in a nonequivalent manner, adhering predominantly to one side, thereby facilitating a diffusion process more akin to surface diffusion (Figure S7B). Importantly, this behavior is also indicative of the stabilization of new Li^+^ sites close to the V_2_O_5_ bilayers in dilute limit, providing a qualitative explanation for the strongly increased Li^+^ storage capacity that was experimentally observed in expanded V_2_O_5_ (maximum capacity of Li_1.48_V_2_O_5_-6C-DA). These observations are further supported by nudged elastic band (NEB) calculations, which confirm the reduced energy barriers along the diffusion pathways; detailed results are provided in Figure S8.

To further investigate the influence of molecular pillars on Li^+^ diffusion behavior, we performed ab initio molecular dynamics (AIMD) simulations on V_2_O_5_ structures containing 2C-DA and 6C-DA pillars. These systems were chosen for computational analysis because of their experimentally demonstrated solid-solution intercalation mechanism of desolvated Li^+^. These simulations were designed to capture both ion transport and dynamic interactions within the hybrid frameworks. To accurately reflect the localized nature of V-3*d* electrons and interlayer interactions, a DFT+U correction (U = 3.25 eV) was applied to vanadium atoms, and van der Waals interactions were included using the DFT-D3 method to account for interactions between V_2_O_5_ layers and organic pillars. Simulations were performed at 600 K in the canonical ensemble (NVT) using a Nosé-Hoover thermostat, which provides sufficient thermal energy to activate relevant diffusion processes. Each trajectory spanned 10 ps with a time step of 1.0 fs, allowing comprehensive sampling of Li^+^ motion and its interactions with both the inorganic framework and molecular pillars.

To quantitatively assess the diffusion behavior of Li^+^ ions within the V_2_O_5_ structures, probability density function (PDF) analysis was performed on the AIMD trajectories (Figure 10A,B). The PDF results, visualized as yellow isosurfaces, represent the spatial distribution of atomic positions over time and provide insight into the dynamic behavior and preferred positions of Li^+^ ions (green spheres) within the simulation box. Consistent with the BVSE simulations (Figure S7), the AIMD results reveal that Li^+^ diffusion in both V_2_O_5_-2C-DA and V_2_O_5_-6C-DA systems predominantly follows a surface-like diffusion mechanism, with Li^+^ ions migrating along pathways adjacent to the V_2_O_5_ bilayers.

**Figure 10:**
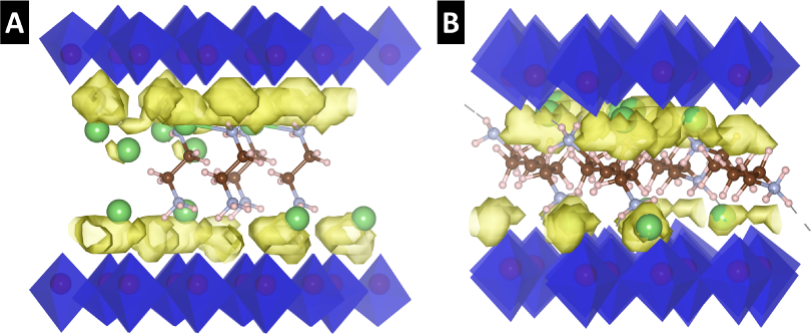
Probability Density Function (PDF) analysis from AIMD trajectories at 600 K for (A) V_2_O_5_-2C-DA and (B) V_2_O_5_-6C-DA. The PDF results (yellow surfaces) represent the distribution of atomic positions over time, providing insights into the diffusion behavior of Li-ions (green spheres) within the system, highlighting the spatial distribution and probability of finding atoms at specific locations within the simulation box. Carbon atoms are represented by brown spheres, nitrogen atoms by light blue spheres, and hydrogen atoms by white spheres. The V_2_O_5_ bilayer is depicted with blue octahedral, emphasizing the structural arrangement. The isosurface value for the PDF is set to 0.001 1/Å^3^, visualizing the probability density of ion movement within the material.

The diffusivity of Li^+^ ions in both systems was evaluated to elucidate the influence of molecular pillars on Li^+^ mobility. For the V_2_O_5_-2C-DA structure, the calculated Li^+^ diffusivity is approximately 3.41 × 10^–6^ cm^2^/s, whereas a higher value of 2.44 × 10^–5^ cm^2^/s is observed for the V_2_O_5_-6C-DA system, confirming the experimentally observed improvement in rate behavior for increased interlayer spacings (Figure 5E). To elucidate the origin of this increase in diffusivity, we performed radial distribution function (RDF) and coordination number (CN) analyses to investigate the local structural environment of Li and V within the pillared V_2_O_5_ frameworks, as shown in Figure 11A–F.

**Figure 11:**
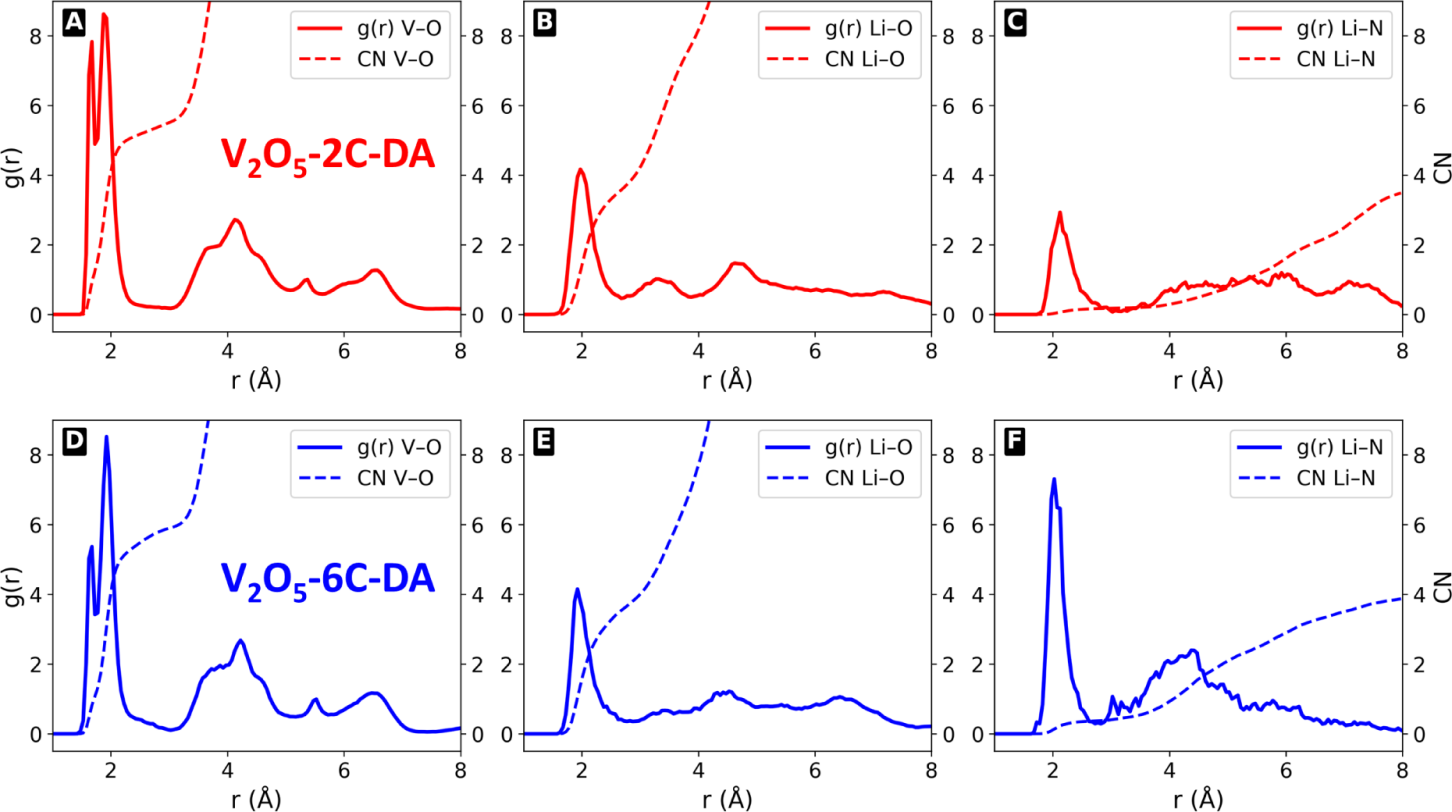
Radial distribution functions (RDF) and coordination number (CN) analyses for (A) V–O, (B) Li–O, and (C) Li–N atom pairs in V_2_O_5_-2C-DA, and (D) V–O, (E) Li–O, and (F) Li–N atom pairs in V_2_O_5_-6C-DA. The V–O RDF shows sharp peaks below and at 2 Å, indicating a well-defined octahedral coordination around the vanadium atoms. In contrast, the Li–O and Li–N RDFs are broader and less structured, with primary peaks around 2 Å, suggesting more disordered and flexible solvation and coordination environments for the Li^+^ ions within the pillared V_2_O_5_ framework.

The V–O pair exhibits a highly ordered structure in both systems, reflected by two sharp RDF peaks: one just below 2 Å and a second around 2 Å (Figure 11A,D). These peaks correspond to strong, well-defined V–O bonds characteristic of edge- and corner-sharing VO_6_ octahedrons. The peak heights are slightly above and below 8 for the 2C-DA system, whereas the first peak height decreases to 5 for the 6C-DA system, indicating reduced octahedral distortion in the presence of the 6C-DA pillar. A broader, weaker peak centered around 4 Å (g­(r) ∼ 1.5) represents a second coordination shell involving more distant oxygen atoms, which remains similar in both systems.

In contrast, the Li–O RDF shows a broader and less structured profile (Figure 11B,E). A primary peak at around 2 Å with g­(r) ∼ 3 corresponds to typical Li–O bond lengths in solvated or intercalated states, followed by broader, less intense peaks around 3.5 Å and 4.5 Å, indicating a more dynamic and disordered solvation environment for Li^+^. The Li–N RDF shows a main peak just above 2 Å, with g­(r) ∼ 3 for 2C-DA and ∼8 for 6C-DA (Figure 11C,F), indicating a stronger interaction between Li^+^ and nitrogen atoms from the diammonium pillars in the 6C-DA system compared to 2C-DA. Beyond 4 Å, the RDF flattens, indicating weak or nondirectional interactions at longer distances.

These analyses reveal that while the V–O sublattice remains highly ordered, the Li^+^ coordination environment is more diffuse, with clear indications of interactions with both oxygen and nitrogen species in the hybrid frameworks. In the 6C-DA system, the stronger Li–N interactions appear to increase diffusivity by competing with Li–O interactions, thereby facilitating ion mobility. Coordination number (CN) analysis further supports these findings: in both systems, vanadium remains coordinated to six oxygen atoms and Li–O coordination numbers range between 3 and 4. However, the Li–N coordination number increases from 0 in 2C-DA to 1 in 6C-DA. The observed increase in Li^+^ diffusivity in the 6C-DA system can thus be attributed to the combined effects of interlayer expansion and enhanced Li–N interactions, which together lower diffusion barriers and promote faster ion transport.

Overall, our study shows that increasing the interlayer spacing by molecular pillars introduces a complex interplay between bonding distances, coordination environments, and diffusion mechanisms. Expansion of the interlayer space enhances connectivity of diffusion pathways, transforming a one-dimensional pathway into a surface-like diffusion mechanism. This mechanism is facilitated by the dynamic and diffuse Li^+^ coordination environment, where interactions with both oxygen and nitrogen species contribute to enhanced mobility. In particular, the introduction of 6C-DA pillars not only increases the interlayer spacing, but also promotes stronger Li–N interactions, which effectively compete with Li–O coordination and lower the energy barriers for ion transport. This combination of interlayer expansion and Li–N interactions results in minimal site preference, ultimately leading to improved ionic mobility. These findings highlight a viable strategy for enhancing Li^+^ conductivity in layered materials through controlled pillar engineering and tuning of interfacial interactions.

## Conclusions

A series of bilayered V_2_O_5_ host materials with well-defined interlayer spacings between 1.0 and 1.9 nm is synthesized using alkyldiamine molecular pillars of different lengths. Detailed structural characterization, including high resolution TEM at length scales relevant to the hosts’ nanoconfinement geometries, reveals the structural properties of pillared V_2_O_5_ in detail. The materials exhibit comparable nanowhisker morphology, intralayer crystal structure, specific surface area, tilted pillar conformation and ionic host-pillar interaction, thus only varying in *c*-lattice parameter. This allows to unambiguously link their electrochemical properties to the variation of nanoconfinement geometry.

The maximum specific capacity for Li^+^ intercalation in the host materials at low rate is between 155 and 193 mAh/g. Subtracting the mass contribution of pillaring molecules that do not provide reversible charge storage capacity, the lithium ion storage capacity per V_2_O_5_ is increased with larger interlayer spacing, with an improvement from approximately 1.0 to 1.5 Li^+^ in the material with the intermediate expansion owing to the stabilization of new storage sites in closer proximity to the V_2_O_5_ bilayers. Furthermore, the rate handling improves with larger interlayer spacings due to a change in diffusion pathway from 1D toward networks of 2D diffusional pathways for Li^+^ when increasing the interlayer spacing of V_2_O_5_ hosts. This demonstrates the possibility of improving ion storage capacity and kinetics in layered host materials by manipulating the nanoconfinement geometry. Operando X-ray diffraction investigation reveals a solid-solution Li^+^ intercalation mechanism with minimal reversible expansion/contraction behavior for V_2_O_5_ hosts with small (V_2_O_5_-2C-DA) and intermediate (V_2_O_5_-6C-DA) interlayer spacing. For V_2_O_5_ with the largest interlayer spacing (V_2_O_5_-12C-DA), an abrupt further expansion is observed. The observations are in line with complementary ECD showing a higher magnitude of electrode thickness changes for V_2_O_5_-12C-DA during (de)­lithiation. This is due to the emergence of the solvent cointercalation mechanism with simultaneous intercalation of up to two electrolyte solvent molecules per one Li^+^, as confirmed by EQCM measurements. It is a nanoconfinement geometry-induced effect, triggered by the large interlayer spacing of V_2_O_5_-12C-DA.

Overall, the work provides systematic insights into the structural properties and associated electrochemistry of pillared V_2_O_5_ materials serving as ion intercalation hosts, which is also of relevance to research into high power energy storage or multivalent ion intercalation cathodes.

## Methods

### Materials Synthesis

The alkyldiamine functionalized V_2_O_5_ was synthesized via hydro- and/or solvothermal synthesis routes in a 95 mL Teflon lined stainless-steel autoclave (BR-100, Berghof). The three alkyldiamines utilized are 1,2-ethylenediamine (C_2_H_8_N_2_, 2C-DA, Sigma-Aldrich), 1,6-hexanediamine (C_6_H_16_N_2_, 6C-DA, ThermoFisher) and 1,12-dodecanediamine (C_12_H_28_N_2_, 12C-DA, ThermoFisher). The molar ratio of alkyldiamine to vanadium used was 1:1. 0.3 g α-V_2_O_5_ powder (ThermoFisher) was added to 50 mL distilled water under constant stirring at room temperature. In the case of 12C-DA, a 1:1 volume ratio of distilled water and ethanol was used due to the low solubility of 12C-DA in water. The respective amounts of alkyldiamines were then added to the above solution with stirring. The pH value of the precursor solution was adjusted to 3 via the addition of 3 mol L^–1^ hydrochloric acid solution (Sigma-Aldrich). The reaction mixtures were heated to 100 °C and held for 12 h, before naturally cooled to room temperature. The products were filtered through a PTFE filter paper (Whatman), washed with distilled water and ethanol, and then dried in an oven at 80 °C for 24 h.

Li_*x*_V_2_O_5_ was synthesized via a sol–gel synthesis approach in accordance with earlier reports by Clites et al.^7^ 2.33 g of LiCl (ThermoFisher) was added to 15 mL deionized water in a glass beaker with constant stirring. Subsequently, 15 mL of 30 wt % H_2_O_2_ aqueous solution (Sigma-Aldrich) was added. 0.5 g α-V_2_O_5_ was then slowly added to the solution under vigorous stirring. The Li:V molar ratio of 10:1 was used to ensure an excess of Li^+^. The solution was stirred for 1 h at room temperature and subsequently heated to 60 °C with constant stirring for 3 h to form a dark red gel. The material was aged for 4 days at room temperature where the product precipitated in the form of a green powder. The product was dissolved in 3 M LiCl solution and hydrothermally treated at 220 °C for 24 h in the autoclave. Lastly, the sample was filtered through a PTFE filter paper, washed with deionized water, and dried at 80 °C for 24 h.

### Physicochemical Characterization

X-ray diffraction of powder samples was carried out using a Bruker D8 Advance diffractometer (Cu Kα radiation, λ = 0.154 nm) in the range of 2°–60° with a step size of 0.025° 2θ at a dwell time of 2 s per angular step. XRD of casted electrodes was performed using a polished Si-crystal holder and was measured in the same 2θ region with a step size of 0.04° 2θ at a dwell time of 14 s per step.

X-ray photoelectron spectroscopy (XPS) was performed on the pristine powders using a SPECS XPS system, equipped with a monochromatic Al Kα X-ray source (*hν* = 1487 eV) and PHOIBOS 150 spectrometer. High-resolution scans of the C 1s, O 1s, V 2p and N 1s transitions were acquired at 400 W, 30 eV pass energy, and 0.1 eV energy step. Calibration of the binding energy was carried out using the adventitious carbon signal in the C 1s region (C–C/C–H) at 284.8 eV as a reference.^55^ The collected spectra were fitted by CasaXPS software using a nonlinear Shirley-type background and a 70% Gaussian/30% Lorentzian line shape.^56^

Thermogravimetric analyses (TGA) were carried out in the temperature range from room temperature to 550 °C at a heating rate of 5 K min^–1^ under constant oxygen flow in aluminum crucibles loaded with ca. 8 mg of material using a TGA209 F1 Libra (Netzsch).

Gas sorption measurements were carried out with an advanced micropore size and chemisorption analyzer (Quantachrome Instruments). The samples were outgassed at 80 °C under vacuum for 22 h before the measurement was carried out under argon gas. The ASiQwin software from Quantachrome was utilized to analyze the data.

### Electron Microscopy

Scanning electron microscopy (SEM) at 5 kV was applied to the powder samples on carbon tape using a Zeiss Crossbeam microscope. A Thermo Fisher/FEI Talos F200X transmission electron microscope (TEM) was utilized to perform bright-field TEM imaging (BFTEM), selected-area electron diffraction (SAED), as well as high-resolution TEM (HRTEM) imaging to prove sample quality and homogeneity, and to in detail characterize the sample morphology, crystal structure, and molecular spacer bilayer separation distance/spacing of the synthesized materials. The microscope, equipped with a high-brightness XFEG gun, was operated at 200 kV and column vacuum, beam current and imaging/capture times (frame integration) were optimized to keep beam damage of the samples at a minimum. The as-synthesized powder samples were dispersed onto TEM support grids (Plano S166-2). To separate the aggregated whiskers, small amounts of the aggregated powder were separated by gently grating two microscope glass slides against each other with the powder placed in between. The materials were dry transferred onto the TEM grids. Larger agglomerates were removed leaving clean scanty material suspended on the grid. Series of SAED patterns, as well as HRTEM images, were sequentially collected and integrated to minimize the applied beam-current density. To analyze the SAED patterns, JEMS software package (by P. Stadelmann, jems-swiss) was employed for pattern simulation and indexing. For precise determination of *c*-axis lattice constants, average radial intensity profiles were determined and the peak positions particularly of the (001) rings were extracted from the second-order derivative of the radial plot profiles.

### Electrode Preparation

The electrodes were fabricated using a slurry with a composition of 80:10:10 wt % active material to carbon black (Super C65, C-NERGY) to polyvinylidene fluoride (PVDF, Solef 6020, Arkema Group) in *N*-methyl-2-pyrrolidone solvent (NMP, anhydrous, Sigma-Aldrich, 2 wt % of PVDF in NMP). The slurries were mixed in a planetary speed mixer (Thinky, ARE-310) for 5 min at 2000 rpm and then cast on a carbon coated aluminum foil (20 μm, battery grade, Welcos), using a laboratory doctor blade with a wet film thickness of 60 μm. The electrode sheets were dried at 80 °C overnight (Binder ED-115 oven), and 12 mm diameter electrodes were cut with a hand puncher (Hohsen). Each disc electrode was weighed to determine the active material mass loading, which was kept low around 0.80 ± 0.20 mg/cm^2^ to minimize the influence of electrode architecture on the electrochemical performance. This was followed by another drying step at 80 °C under vacuum for 16 h before transfer to the glovebox.

### Electrochemical Characterization

Electrochemical characterization was carried out using 2032 type coin cells (Hohsen), which were assembled in an argon-filled glovebox (MBraun, O_2_ and H_2_O < 0.1 ppm). They contained a stack composed of the V_2_O_5_ electrodes on aluminum current collectors, a glass fiber separator (19 mm diameter, GF/A, Whatman), a 12 mm diameter lithium disc (500 μm thickness, Honjo), a stainless-steel spacer and a spring. As electrolyte, 90 μL of 1.0 M LiPF_6_ in ethylene carbonate/diethyl carbonate is used (EC:DEC, 3:7 volume ratio, Powerlyte). All electrochemical experiments were performed in climatic chambers (Binder) at 20 °C with potentiostats from Biologic (VMP3, VMP-3e, VMP-300). Cyclic voltammetry (CV) and galvanostatic cycling were conducted in the potential window of 2.0–4.0 V vs the Li^+^/Li. For long-term cycling, a specific current of 500 mA/g was employed. To test the rate capability, specific currents of 20, 50, 100, 200, 500, 1000, 5000 mA/g were applied for 5 cycles each and the last 5 cycles were again run at 20 mA/g. All normalizations are with respect to combined mass of V_2_O_5_ with molecular pillar, unless explicitly stated otherwise.

Electrochemical impedance spectroscopy (EIS) was carried out in custom-built three-electrode cells based on a polyether ether ketone body and titanium pistons (cell described in ref 57. In addition to the V_2_O_5_-based working electrodes, the cells consisted of a Li metal counter electrode (12 mm disc, as described above) and a separate small piece of Li metal as the quasi-reference electrode that was contacted by a titanium screw from the side channel. EIS spectra were recorded at various potentials using the staircase potential electrochemical impedance spectroscopy (SPEIS) setting. Spectra were recorded in the frequency range of 200 kHz to 100 mHz, with a voltage amplitude of 10 mV. The potential was changed from OCV to 2 V vs Li^+^/Li in 100 mV increments, with a 30 min holding step at each potential prior to recording an impedance spectrum.

### Electrochemical Operando Experiments

For operando XRD experiments, slurries of the V_2_O_5_-based materials (80 wt % active material, 10 wt % carbon black, 10 wt % PVDF in NMP, ca. 2 mg/mL) were coated on Ti mesh current collectors (12 mm diameter) by drop casting (mass loadings ca. 7–9 mg/cm^2^). These electrodes were assembled together with Li metal negative electrodes, a glass fiber separator (GF/D, Whatman) and 1.0 M LiPF_6_ in EC:DEC in modified coin cells with 6 mm openings in the top and bottom parts, which were sealed with Kapton tape. Measurements were carried out in transmission geometry with a STADI-p diffractometer (STOE) equipped with a coin cell holder and a molybdenum X-ray source (Mo K_α_ radiation, λ = 0.07093 nm). Using a mobile potentiostat (SP-300, Biologic), the cells were cycled at a specific current of 50 mA/g over five cycles.

Electrochemical dilatometry (ECD) was conducted using an ECD-4-nano dilatometer (EL-CELL). For electrode preparation, self-standing electrodes of the V_2_O_5_-based materials were used, composed of 80 wt % active material, 10 wt % carbon black, and 10 wt % of polytetrafluoroethylene (PTFE, 60 wt % dispersion in H_2_O, Sigma-Aldrich).The electrode dry thickness was ca. 50–60 μm. The ECD cell was rested at open circuit potential for 6 h prior to electrochemical measurements at 23 °C in a temperature-controlled chamber.

Electrochemical quartz crystal microbalance (EQCM) experiments were carried out with an AWS X1 device from AWSensors with the temperature control unit of the QCM set to 23 °C. Electrodes were prepared by spray-coating of dilute slurries (80 wt % active material, 10 wt % carbon black, 10 wt % PVDF in NMP, ca. 0.25 mg PVDF per mL of NMP) onto the quartz sensors (5 MHz, 14 mm, Ti/Au, AWSensors) using an airbrush gun (Gaahleri). This resulted in thin-films with low active mass loadings of ca. 20 μg/cm^2^, ensuring rigid properties of the coatings (Figure S5). The mass change was estimated using the Sauerbrey equation:^52^

Δm=−C·Δf

where Δm is the mass change, C is the sensitivity constant of the used quartz crystal (17.98 ng/Hz) and Δf is the frequency change.

### Computational Details

Density functional theory (DFT) was employed to study the influence of the *d*-spacing on the Li^+^ diffusion barriers in bilayered V_2_O_5_. First, a benchmark of DFT functionals was conducted using the generalized gradient approximation (GGA) with the Perdew–Burke–Ernzerhof (PBE)^58^ functional, the nonempirical strongly constrained and appropriately normed (SCAN)^59^ meta-GGA functional, and the hybrid Heyd-Scuseria-Ernzerhof (HSE) functional (α = 0.25)^60,61^ within the projected augmented wave (PAW) method^62,63^ as implemented in the Vienna ab initio simulation package (VASP). The third generation (D3)^64^ semiempirical van der Waals corrections proposed by Grimme and the revised Vydrov–van Voorhis (rVV10) nonlocal correlation functional,^65,66^ respectively, were integrated into the structure optimizations. In addition, the influence of including Hubbard-type correction to accurately depict the behavior of localized d-electrons on the structural parameters was tested with the datadriven Hubbard U values of U_PBE_ = 3.25 ^67^ and U_SCAN_ = 1.0.^68^ The benchmarking of the DFT functional employed in this study is presented in Table S1 of the Supporting Information. The plane-wave cutoff energy was set at 520 eV, with Monkhorst–Pack (MP)^69^ k-point meshes of 3 × 3 × 3 representing the Brillouin zone. Activation barriers and minimum energy paths for carrier hopping were determined by the climbing image nudged elastic band (cNEB) method.^70,71^ The diffusion path was first constructed by linear interpolation of atomic coordinates between initial and final states with five distinct images, followed by relaxation until the forces on all atoms were below 0.05 eV Å^–1^. In the cNEB computations, the total energies were assessed using the SCAN+rVV10 functional, without incorporating any U corrections. Large supercells were used to ensure ion isolation from periodic images (ions separated by at least 10 Å). Initial atomic configurations were obtained from the Materials Project (MP) database.^72^

Ab initio molecular dynamics (AIMD) simulations were performed in the NVT ensemble with a Nosé-Hoover thermostat at 600 K, using a time step of 1.0 fs for trajectories up to 10 ps. The initial structure was constructed as a V_2_O_5_ supercell with 12 formula units, containing 5 Li atoms and 2 molecular pillars. A plane-wave cutoff energy was set at 450 eV, and Brillouin zone sampling was restricted to the Γ point. The Li^+^ diffusivity was calculated from the AIMD trajectories using the pymatgen library,^73,74^ which is based on mean squared displacement (MSD) analysis.

## Supplementary Material



## Data Availability

Experimental data used in this work (including transmission electron microscopy, diffraction, spectroscopy, and electrochemical cycling) are made available on the Zenodo repository (https://zenodo.org) at https://zenodo.org/doi/10.5281/zenodo.12799606. All electronic structure calculations used in this work are made available under the Creative Commons Attribution license (CC BY 4.0) on the NOMAD repository (https://nomad-lab.eu) at https:/doi.org/10.17172/NOMAD/2024.07.23-1.

## Notes

Additional structural characterization, galvanostatic cycling, ex situ XRD, kinetic analysis of electrochemical charge storage by b-values, extended EQCM data, simulated bond valence site energy profiles and diffusion pathways as a function of V_2_O_5_ interlayer spacing, and a table of simulated lattice constants (PDF)
