# Supplementary material for: Nanoconfinement Geometry of Pillared V2O5 Determines Electrochemical Ion Intercalation Mechanisms, Storage Sites, and Diffusion Pathways
Source: ACS Nano. 2025 Jul 14;19(29):26904–19. doi: 10.1021/acsnano.5c08169 (PMC12312152; doi:10.1021/acsnano.5c08169)
Supplement: Supplementary file 1 [file nn5c08169_si_001.pdf]

Supporting Information

# Nanoconfinement geometry of pillared $V_2O_5$ determines electrochemical ion intercalation mechanism, storage sites and diffusion pathways

Jameela Karol,[1,2] Charles O. Ogolla,[3] Mohsen Sotoudeh,[1,2,4] Manuel Dillenz,[4] Maciej Tobis,[1,2] Ellen Vollmer,[1,2] Yoga T. Malik,[1,2] Maider Zarrabeitia,[1,2] Axel Groß,[1,4] Benjamin Butz,[3] Simon Fleischmann[1,2*]

1 Helmholtz Institute Ulm (HIU), Helmholtzstr. 11, 89081 Ulm, Germany

2 Karlsruhe Institute of Technology (KIT), 76021 Karlsruhe, Germany

3 University of Siegen, Micro- and Nanoanalytics Group, 57076 Siegen, Germany

4 Institute of Theoretical Chemistry, Ulm University, Ulm, 89081 Germany

* Corresponding author's email: simon.fleischmann@kit.edu

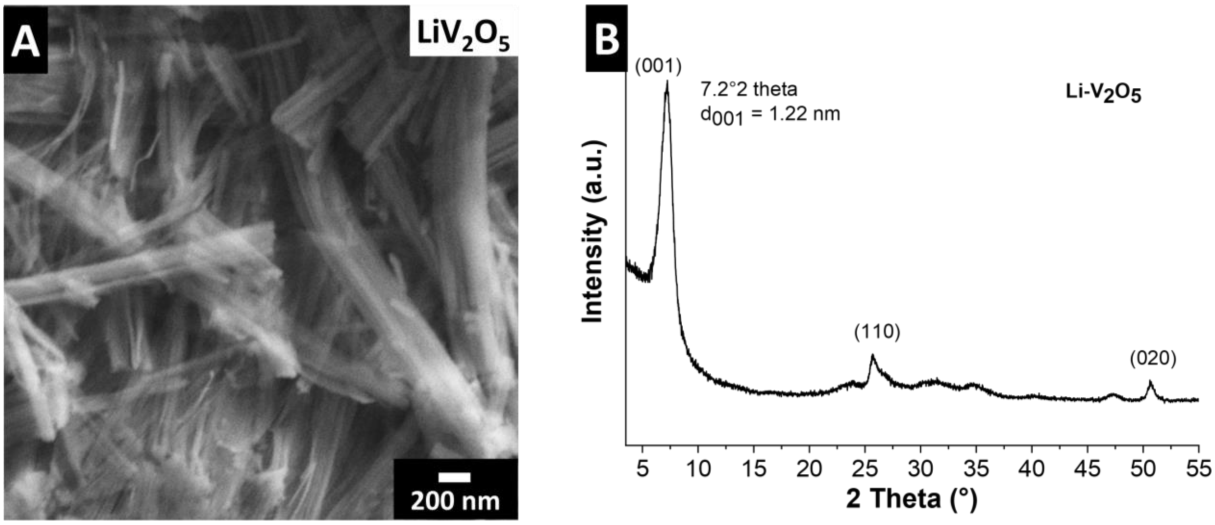


**Fig. S1**: Structural characterization of hydrothermally synthesized Li-$V_2O_5$ sample. (A) Scanning electron micrograph and (B) X-ray diffractogram.

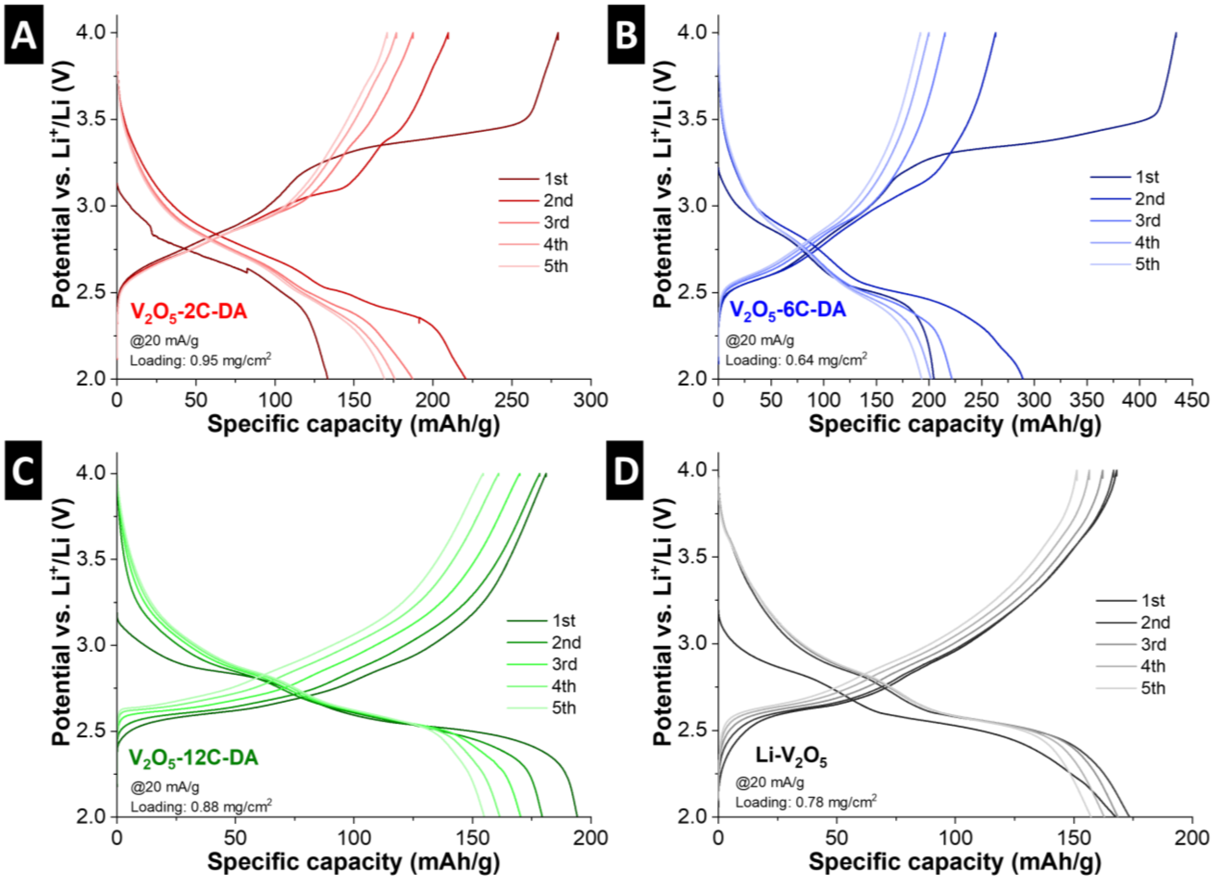


**Fig. S2**: First five consecutive galvanostatic cycles at a specific current of 20 mA/g of (A) $V_2O_5$-2C-DA, (B) $V_2O_5$-6C-DA, (C) $V_2O_5$-12C-DA, and (D) Li-$V_2O_5$. All measurements are conducted in coin cells versus Li metal electrodes in 1 M $LiPF_6$ in EC/ DEC (30:70 vol.%) electrolyte.

Irreversible capacity losses are observed, particularly in the first cycle of $V_2O_5$-2C-DA and $V_2O_5$-6C-DA, likely related to irreversible changes such as the reaction of remaining amine groups ($NH_2$) molecules confined in the interlayer space (as identified by XPS). These may also partially dissolve over prolonged cycling (see EQCM section). Interestingly, the highest first cycle efficiency is observed for $V_2O_5$-12C-DA, where a different lithiation mechanism is observed (see operando XRD section). Reducing the first-cycle irreversibility of pillared $V_2O_5$ will be important for practical implementation and is the topic of future work focused on performance optimization.

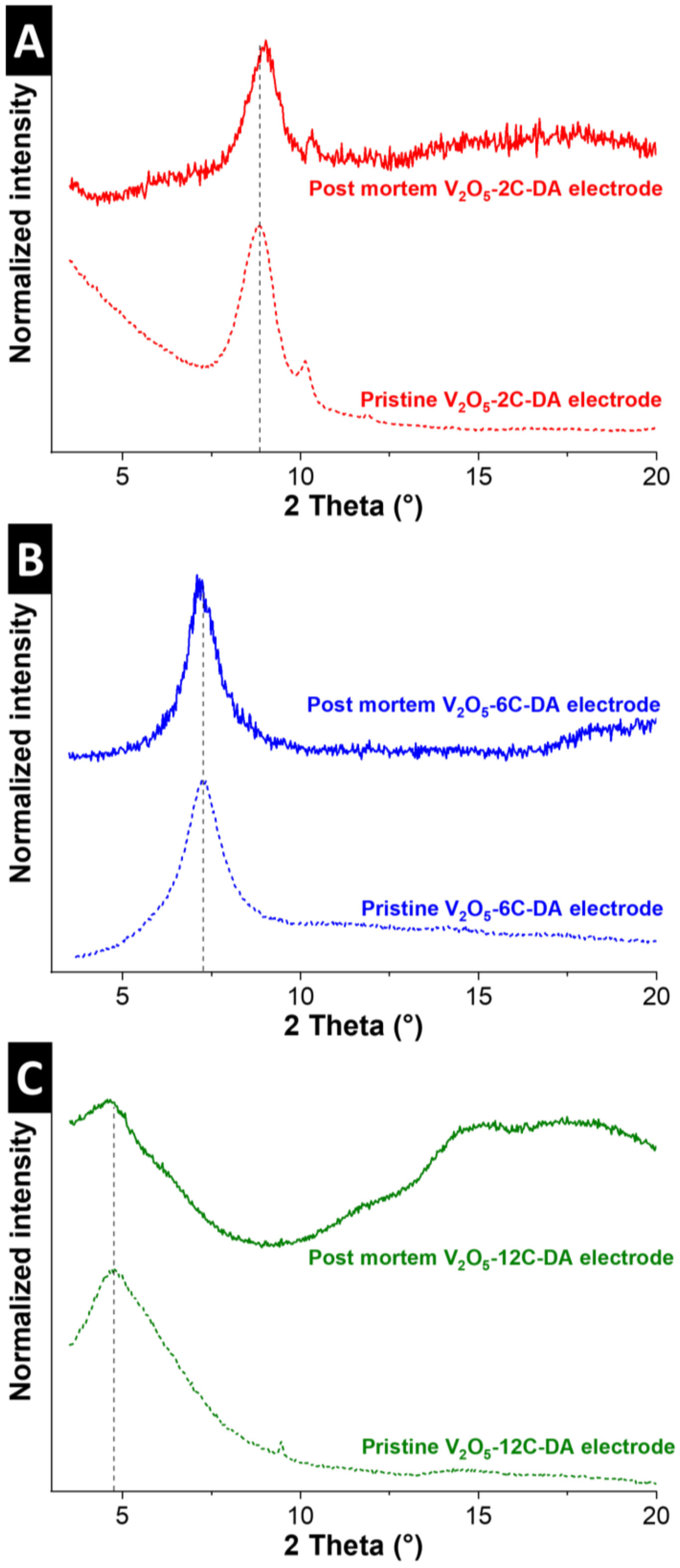


**Fig. S3**: XRD pattern of pristine and cycled electrodes ("post mortem", after 50 charge/discharge cycles at a rate of 500 mA/g) of (A) $V_2O_5$-2C-DA, (B) $V_2O_5$-6C-DA, and (C) $V_2O_5$-12C-DA.

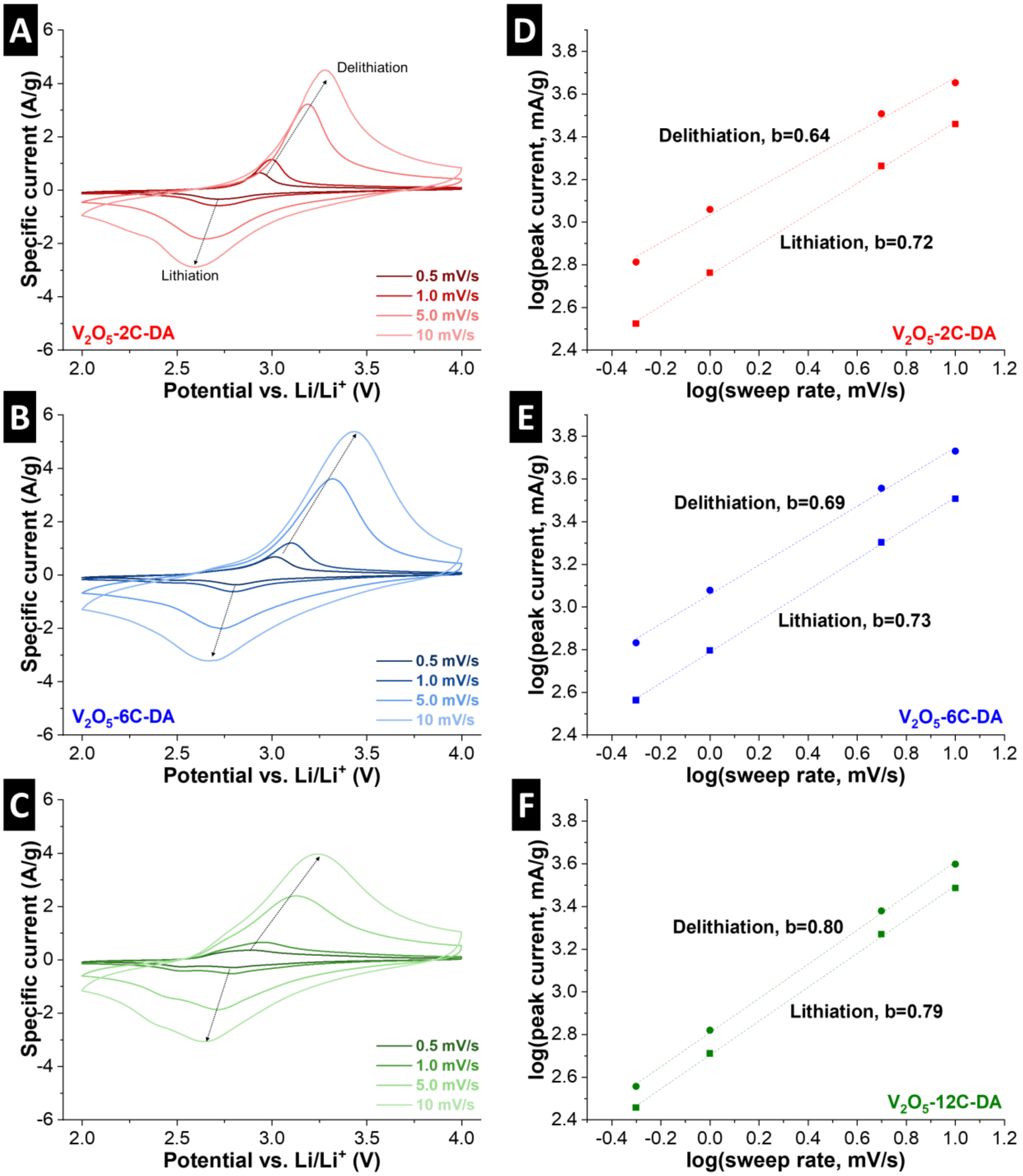


**Fig. S4**: Kinetic study using "b-value" analysis. CVs at varying rates for (A) $V_2O_5$-2C-DA, (B) $V_2O_5$-6C-DA, and (C) $V_2O_5$-12C-DA. (D-F) Corresponding logarithmic plots of cathodic and anodic peak currents versus logarithmic sweep rates, including linear fits and slope ("b-value").

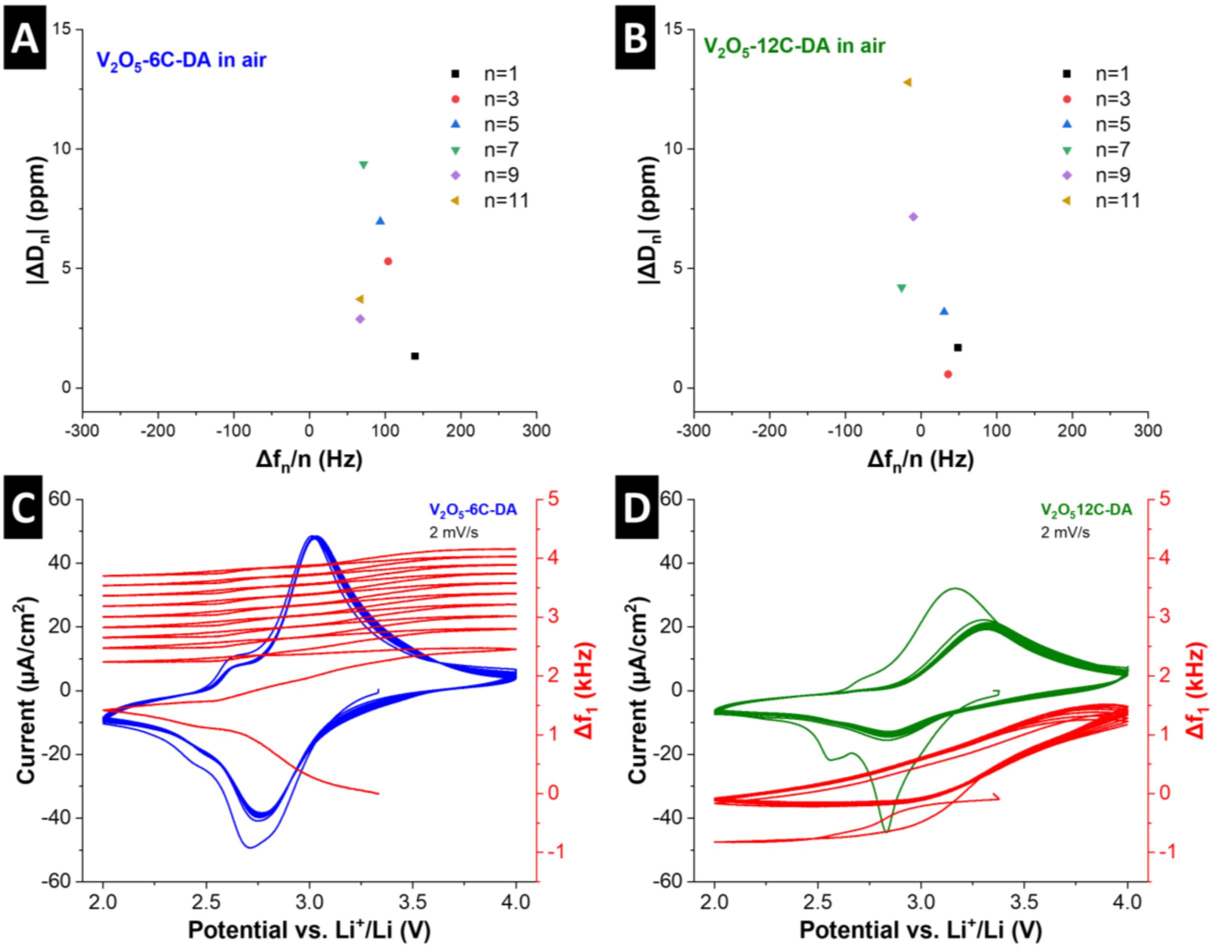


**Fig. S5**: (A-B) Evaluation of dry $V_2O_5$-based coatings on the quartz resonators in air, indicating that the coatings show very small dissipative energy losses (ΔD) at all monitored overtones (up to n=11) that stay below ca. $10^{-5}$. (C-D) Electrochemical quartz crystal microbalance measurements of $V_2O_5$-6C-DA and $V_2O_5$-12C-DA over up to 10 cycles.

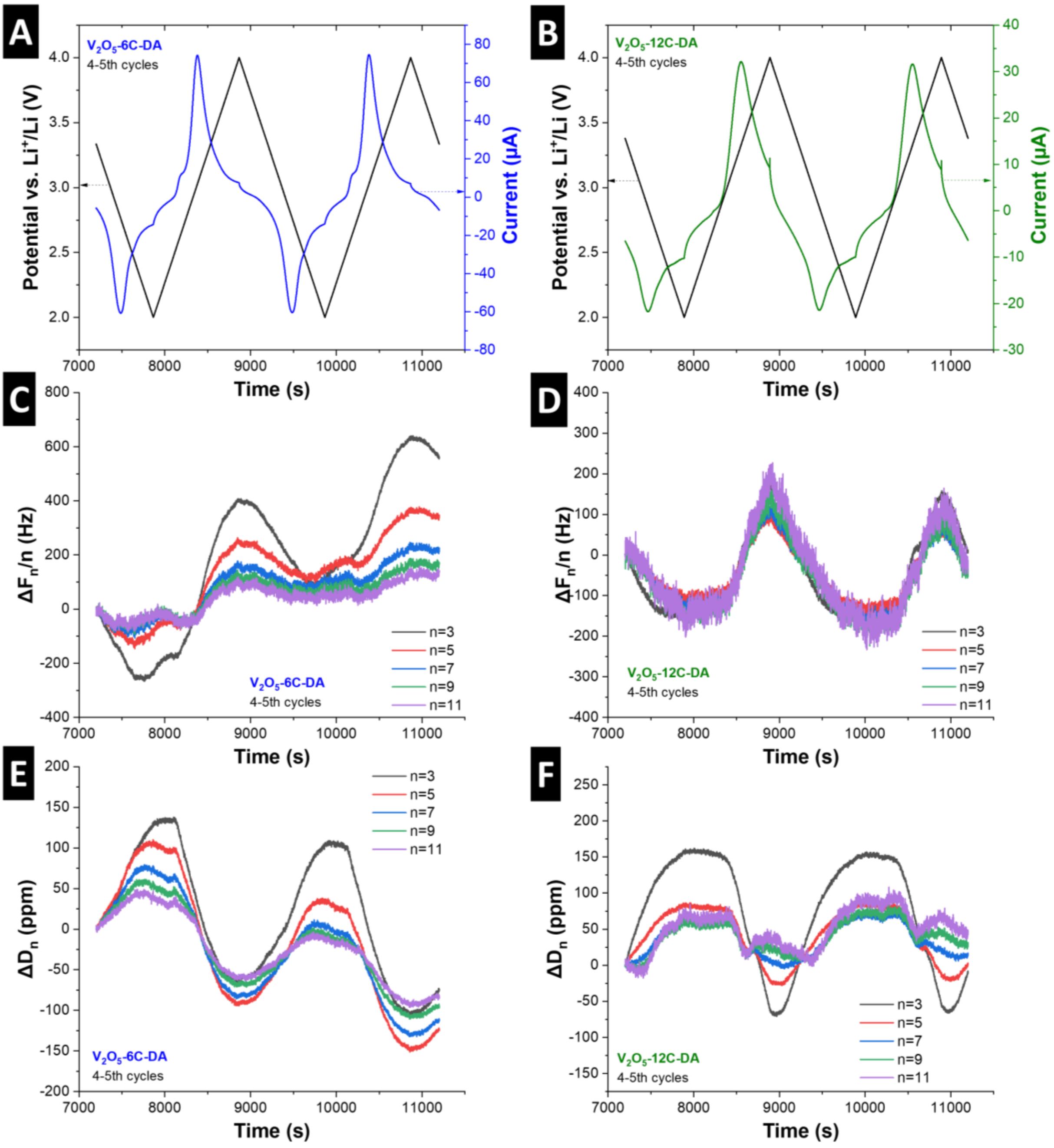


**Figure S6**: Analysis of overtones n (n = 3, 5, 7, 9, 11) during EQCM measurements. (A-B) Electrochemical voltammetry data, (C-D) overtone frequency changes, and (E-F) dissipation of $V_2O_5$-6C-DA and $V_2O_5$-12C-DA electrode coatings.

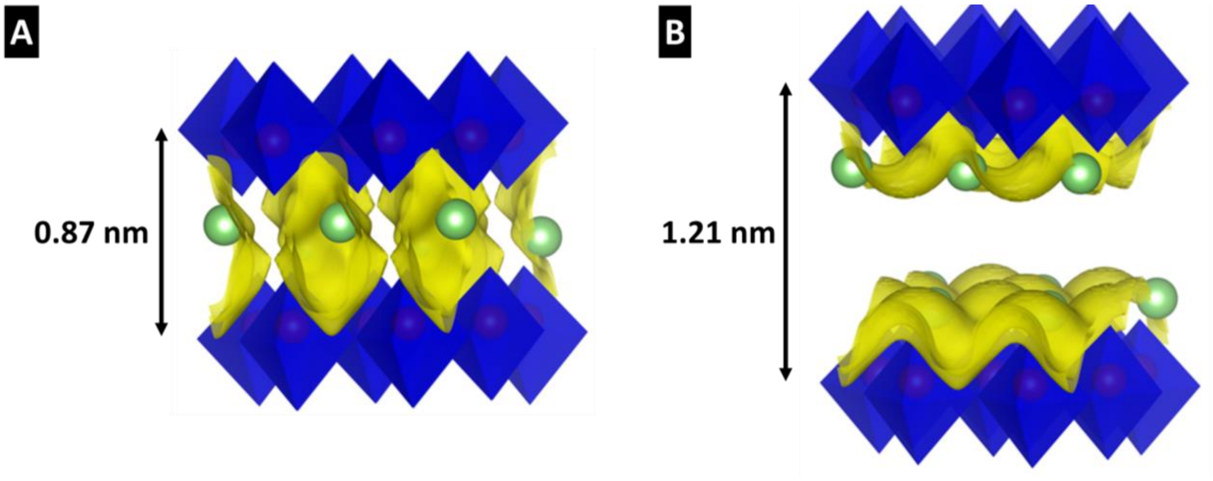


**Figure S7**: Simulated Bond Valence Site Energy (BVSE) profiles comparing the pristine compound with the compound following an increase in interlayer spacing. Green spheres represent lithium atoms, while blue octahedrons depict vanadium atoms coordinated by oxygen in an octahedral fashion. The yellow surfaces illustrate pathways for $Li^+$ diffusion. The isovalue is set to 0.08 e/Å$^3$.

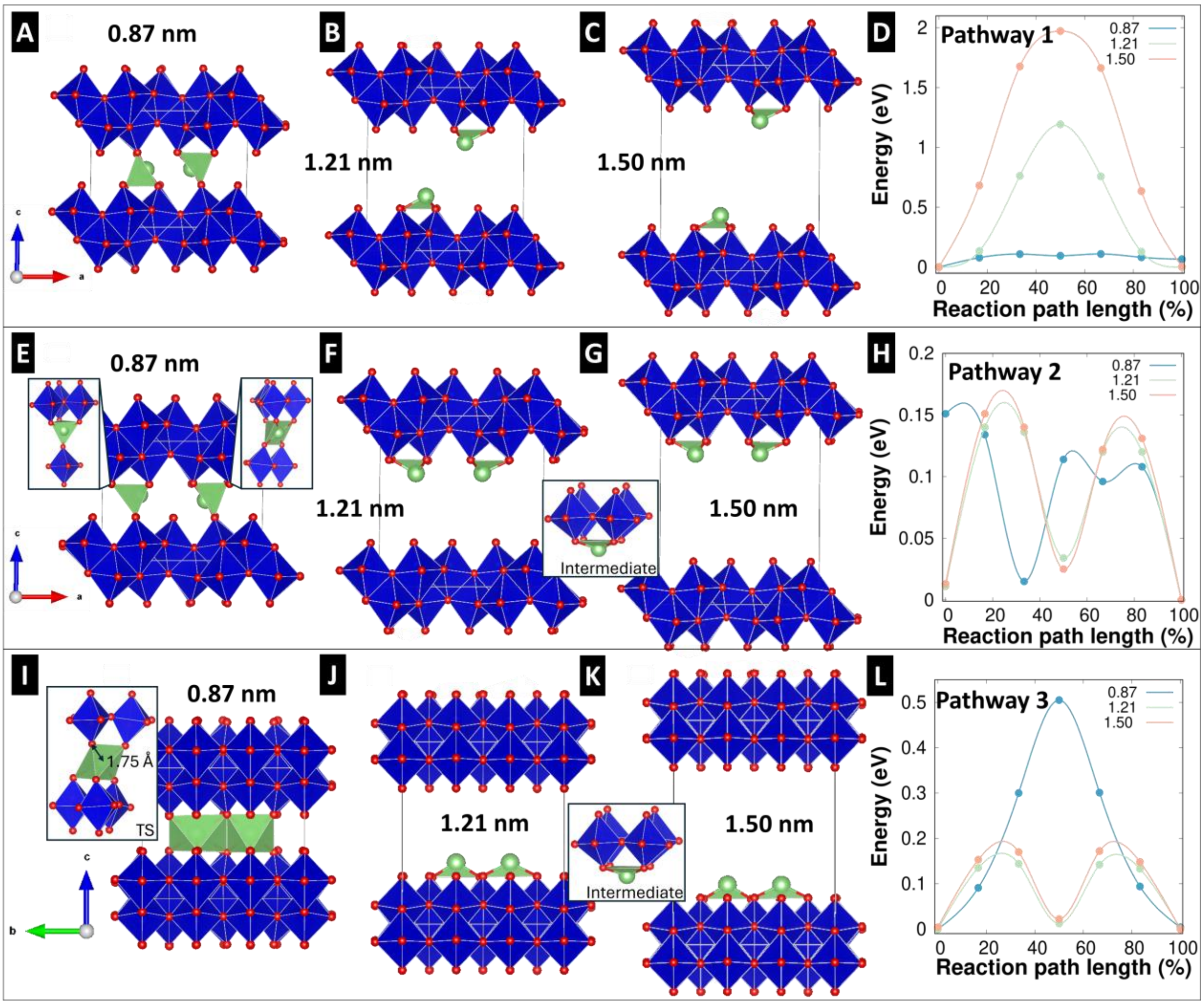


**Figure S8**: Side views of the initial and final states for increasing d-spacings from 0.87 to 1.21 to 1.50 nm, with associated energy barriers for Li+ diffusion plotted against the reaction path coordinate, for diffusion pathways 1 (A-D), 2 (E-H), and 3 (I-L). Results obtained via periodic DFT calculations in combination with NEB method. The insets in (E) highlight the difference in coordination environment between initial 4-fold coordination (left) and final 5-fold coordination (right). The inset in (F) and (G) shows the coordination of the intermediate site of the diffusion pathway. The inset in (I) shows the transition state configuration characterized by a 5-fold coordination of the Li ion. The short bond distance of 1.75 Å is much shorter than the sum of the ionic radii of $O^{2-}$ (1.4 Å) and $Li^+$ (0.76 Å) and leads to substantial diffusion barriers of $E_a$ = 0.51 eV for a d-spacing of 8.6 Å. The inset in (J) and (K) shows the coordination of the intermediate site of the diffusion pathway.

Investigation is conducted into the mechanism of $Li^+$ diffusion using Nudged Elastic Band (NEB) calculations within the framework of periodic DFT. The focus is on the effect of interlayer spacing in a dilute limit without pillar molecules. Initially, the Li-free $V_2O_5$ structure with a d-

spacing of 0.87 nm is employed to assess the diffusion mechanisms and their activation barriers. Subsequently, the diffusion mechanisms and barriers for d-spacings of 1.21 nm and 1.50 nm are examined. Three distinct diffusion pathways are identified. Diffusion pathway 1 connects two pyramidal $Li^+$ sites, each initially exhibiting a 5-fold coordination environment, with three oxygen atoms on one side and two on the opposite side of the layers (**Fig. S8A-C**). Note that both sites are more prominently bound (three vs. two coordinating oxygen atoms) to opposite $V_2O_5$ bilayers. As the interlayer spacing increases, the two oxygen atoms from the upper layer are no longer capable of forming bonds, reducing the coordination number from 5-fold to 3-fold, as illustrated in **Fig. S8B-C**. In addition, the diffusion of $Li^+$ along this pathway entails traversing the interlayer space in between the $V_2O_5$ bilayers in which an effective coordination of $Li^+$ ion is no longer maintained (**Fig. S9**). This, in turn, leads to the formation of high diffusion barriers (>1 eV), which excludes this pathway from contributing to $Li^+$ conductivity (**Fig. S8D**).

Diffusion pathway 2, which commences at an initially 4-fold coordination site, coordinated by three oxygen atoms in the upper layer and only one oxygen atom in the bottom layer (**Fig. S8E**), demonstrates an invariant activation barrier (0.14-0.15 eV) due to a change in diffusion mechanism when the interlayer spacing increased (**Fig. S8H**). For an interlayer spacing of 0.87 nm, the initial site is 0.15 eV higher in energy than the final site and the diffusion pathway involves two intermediate sites. The first intermediate site is equivalent to the final 5-fold coordination, however, predominantly bound to the bottom layer and thus corresponds to traversing the empty space in between. The second intermediate is analogous to that observed in the first pathway, which exhibits planar 4-fold coordination. However, as the interlayer spacing increases, the diffusion mechanism undergoes a transformation (**Fig. S8F-G**). This is due to the inability of oxygen atoms from the opposing side of the layer to serve as effective coordinators and the corresponding large barrier for $Li^+$ to jump to the opposing side, thus minimizing the site preference of the initial and final states. These findings demonstrate that $Li^+$ diffusion along a single site of the $V_2O_5$ bilayer is facilitated by a change in its operational mode as the interlayer spacing increases.

Diffusion pathway 3, which involves surface-like diffusion along pyramidal 5-fold coordination sites predominantly bound to the same layer (**Fig. S8I-L**), also shifts to a mechanism similar to the second pathway. In the case of the pristine structure with an interlayer spacing of 0.87 nm, the diffusion pathway involves a transition state with short bond distances (d=1.75 Å) to

oxygen atoms from the opposing layer (**Fig. S8I**) and high activation barriers of 0.51 eV. When the interlayer spacing increases, a 4-fold intermediate site emerges instead, leading to a shift in mechanism and low diffusion barriers (0.14-0.17 eV) (**Fig. S8L**).

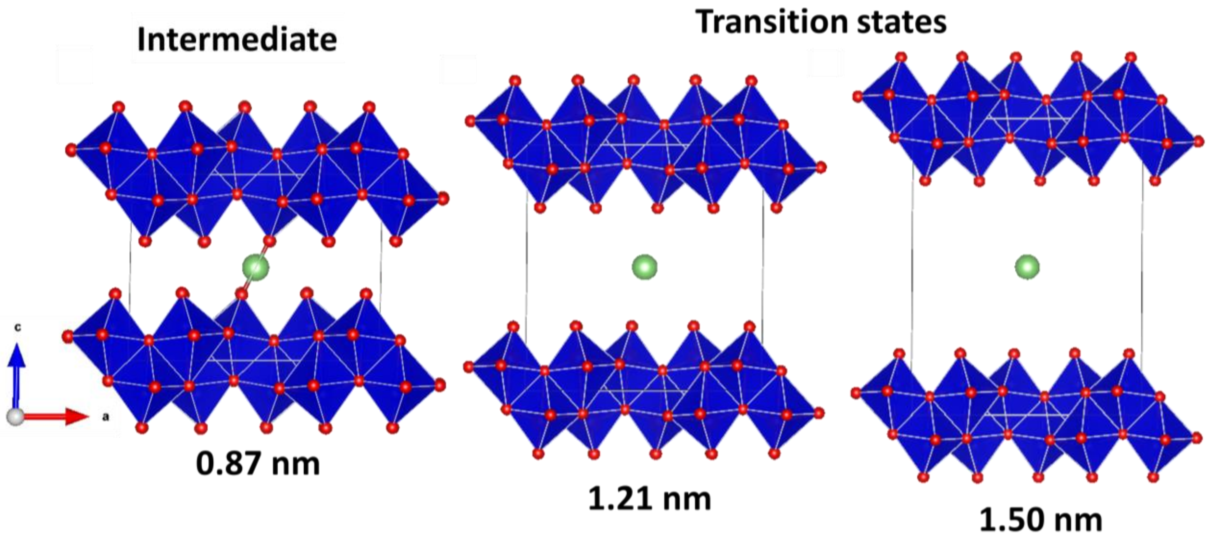


**Fig. S9**: Diffusion pathway 1: Intermediate state (for 0.87 nm) and transition states (for 1.21 and 1.50 nm).

**Tab. S1**: The lattice constants of bilayered $V_2O_5$ without any confined interlayer species, determined employing various exchange-correlation functionals. The addition of the D3 dispersion corrections, as well as the U value of the Hubbard-type energy corrections, is indicated when used. The effect of long-range dispersion interactions is most prominently attributed to a contraction of the c lattice parameter, which corresponds to the d-spacing in the structure.

| $E_{xc}$ | a [Å] | b [Å] | c [Å] | α [˚] | β [˚] | γ [˚] |
|---|---|---|---|---|---|---|
| PBE+U ($U_{eff}$=3.25 eV) | 11.73 | 11.00 | 10.97 | 90.0 | 85.87 | 90.0 |
| PBE+U+D3 ($U_{eff}$=3.25 eV) | 11.69 | 10.98 | 8.89 | 90.0 | 88.69 | 90.0 |
| SCAN | 11.63 | 10.79 | 8.86 | 90.0 | 87.70 | 90.0 |
| SCAN+U ($U_{eff}$=1.0 eV) | 11.64 | 10.84 | 10.26 | 90.0 | 84.77 | 90.0 |
| SCAN+D3 | 11.63 | 10.77 | 8.70 | 90.0 | 87.81 | 90.0 |
| SCAN+rVV10 | 11.63 | 10.79 | 8.66 | 90.0 | 89.01 | 90.0 |
| SCAN+rVV10+U ($U_{eff}$=1.0 eV) | 11.65 | 10.83 | 9.03 | 90.0 | 83.27 | 90.0 |
| HSE06+D3 | 11.61 | 10.74 | 8.46 | 90.0 | 85.33 | 90.0 |
